# The efficacy and safety of hydroxychloroquine for COVID-19 prophylaxis: A systematic review and meta-analysis of randomized trials

**DOI:** 10.1371/journal.pone.0244778

**Published:** 2021-01-06

**Authors:** Kimberley Lewis, Dipayan Chaudhuri, Fayez Alshamsi, Laiya Carayannopoulos, Karin Dearness, Zain Chagla, Waleed Alhazzani, for the GUIDE Group

**Affiliations:** 1 Department of Medicine, McMaster University, Hamilton, Canada; 2 Department of Health Research Methods, Evidence, and Impact, McMaster University, Hamilton, Canada; 3 Department of Internal Medicine, College of Medicine and Health Sciences, United Arab Emirates University, Al Ain, United Arab Emirates; 4 St. Joseph’s Healthcare Hamilton, Hamilton, Canada; University of British Columbia, CANADA

## Abstract

**Background:**

Populations such as healthcare workers (HCW) that are unable to practice physical distancing are at high risk of acquiring Coronavirus disease-2019 (COVID-19). In these cases pharmacological prophylaxis would be a solution to reduce severe acute respiratory syndrome coronavirus-2 (SARS CoV-2) transmission. Hydroxychloroquine has *in vitro* antiviral properties against SARS CoV-2. We therefore sought to determine the efficacy and safety of hydroxychloroquine as prophylaxis for COVID-19.

**Methods and findings:**

We electronically searched EMBASE, MEDLINE, the Cochrane COVID-19 Register of Controlled Trials, Epistemonikos COVID-19, clinicaltrials.gov, and the World Health Organization International Clinical Trials Registry Platform up to September 28^th^, 2020 for randomized controlled trials (RCTs). We calculated pooled relative risks (RRs) for dichotomous outcomes with the corresponding 95% confidence intervals (CIs) using a random-effect model. We identified four RCTs (n = 4921) that met our eligibility criteria. The use of hydroxychloroquine, compared to placebo, did not reduce the risks of developing COVID-19 (RR 0.82, 95% CI 0.65 to 1.04, moderate certainty), hospitalization (RR 0.72, 95% CI 0.34 to 1.50, moderate certainty), or mortality (RR 3.26, 95% CI 0.13 to 79.74, low certainty), however, hydroxychloroquine use increased the risk of adverse events (RR 2.76, 95% CI 1.38 to 5.55, moderate certainty).

**Conclusion:**

Although pharmacologic prophylaxis is an attractive preventive strategy against COVID-19, the current body of evidence failed to show clinical benefit for prophylactic hydroxychloroquine and showed a higher risk of adverse events when compared to placebo or no prophylaxis.

## Introduction

Constraining the propagation of severe acute respiratory syndrome coronavirus-2 (SARS CoV-2) is of the utmost importance to reduce the global burden of this pandemic. While the majority of the population is urged to adhere to physical distancing, it is not possible in all situations. For instance, household transmission rates are quoted to be upwards of 16% [1]. Healthcare workers (HCW) are also at increased risk of acquiring Coronavirus disease-2019 (COVID-19) as they forgo physical distancing and continue to provide patient care, leading to a high number of exposures and infections. China reported that HCW constituted up to 3.8% of laboratory confirmed COVID-19 cases, out of which 14.8% developed critical disease [2]. However, that number is on the rise and reports from Italy state that up to 9% of COVID-19 cases occurred in HCW [3]. Currently, personal protective equipment (PPE) alongside other infection control precautions are the mainstay preventive measures. However, there is a global shortage of PPE, which mandates finding alternative solutions to protect high-risk groups.

A potential solution to reduce the risk of infection is pharmacologic prophylaxis. Antiviral prophylaxis in HCW has been used for other viral illnesses such as the human immunodeficiency virus [4]. A prophylactic agent should be safe, convenient to use (to enhance compliance), and effective. Hydroxychloroquine and chloroquine may theoretically possess all these elements.

Chloroquine and its analogue hydroxychloroquine are commonly used for malaria prophylaxis and treatment, while hydroxychloroquine is often used in some rheumatologic conditions such as systemic lupus erythematosus. Both have the potential to be an effective treatment of COVID-19 through two main mechanisms. First, they inhibit SARS CoV-2 entry to human cells and prevent its replication [5, 6]. Second, they may prevent the most fulminant forms of COVID-19 including cytokine release syndrome (CRS), as they inhibit the activation and production of several cytokines that are characteristically elevated in patients with COVID-19 [6].

While randomized controlled trials (RCTs) have evaluated hydroxychloroquine as a prophylactic agent to protect against SARS CoV-2 infections, their results have not been systematically summarized. Herein, we report a systematic review and meta-analysis examining the efficacy and safety of hydroxychloroquine as prophylaxis for COVID-19.

## Methods

### Research question

In adults (≥18 years old) who had exposure to or at high risk of COVID-19, does the use of prophylactic hydroxychloroquine or chloroquine, versus placebo, reduce the risk of SARS CoV-2 transmission, mortality, and hospitalization?

### Trial selection

#### Eligibility criteria

We included RCTs, while pseudo or quasi-randomized and non-randomized studies were excluded. The trial population included adults (≥18 years old) who are SARS CoV-2 negative [via polymerase chain reaction (PCR) or asymptomatic] at the time of enrolment, and were either in contact with an individual positive for SARS CoV-2, or at high risk of exposure to SARS CoV-2 such as HCWs. The intervention group received oral hydroxychloroquine or chloroquine, at any dose, frequency, or duration, as either pre or post-exposure prophylaxis. The control group did not receive quinines or received placebo for blinding. Eligible trials reported on at least one of the following outcomes: SARS CoV-2 infection; severity of COVID-19 symptoms; duration of COVID-19 symptoms; hospitalization; mortality at longest follow-up; admission to the intensive care unit (ICU); medication compliance; and adverse events.

### Search method for identification of trials

#### Electronic searches

An experienced professional medical librarian designed the search strategy (KD). We electronically searched EMBASE, MEDLINE, the Cochrane COVID-19 Register of Controlled Trials (CENTRAL), and Epistemonikos COVID-19 database every two weeks from inception to September 28^th^, 2020. The search strategies are presented S1–S4 Tables. The search was not restricted by publication status or language. A CADTH (Canadian Agency for Drugs & Technologies in Health) database RCT search strategy was used to include randomized trials exclusively [7]. We also searched ongoing or unpublished trials in clincialtrials.gov and the World Health Organization (WHO) International Clinical Trials Registry Platform (ICTRP) up to September 28^th^, 2020.

### Data collection and analysis

#### Selection of trials

Four reviewers (KL, FA, DC, and LC) screened titles and abstracts, independently and in duplicate, to identify potentially eligible trials, then evaluated the full-texts of potentially eligible trials. Reviewers also screened the reference list of review articles and other systematic reviews for additional trials. Disagreements between reviewers were resolved through discussion. We also contacted the trial authors for further information when required.

#### Data extraction and management

KL, FA and DC, independently used a pre-designed and piloted data abstraction form. In duplicate, reviewers extracted data on: trial eligibility criteria and patients demographic data including age, sex, comorbidities, exposure risks, PPE use; data on hydroxychloroquine or chloroquine dose, route of administration, timing of initiation, and duration of treatment were recorded; the use of placebo or usual care; outcomes (listed above); and relevant information to determine risk of bias. Disagreements were resolved by discussion and consensus.

### Risk of bias

Two reviewers (KL and DC) independently assessed trials for risk of bias using the Revised Cochrane risk-of-bias tool for randomized trials [8]. The overall risk of bias for each trial was categorized as low if the risk of bias was low in all domains, some concern if the risk of bias was deemed to have some concern in at least one domain and with no high risk of bias in any domain, or high if the risk of bias was high in at least one domain per the risk of bias tool. We resolved disagreements by discussion and consensus.

### Measurement of treatment effect

We conducted all analyses using RevMan software (Review Manager, version 5.3. Copenhagen: The Nordic Cochrane Centre, The Cochrane Collaboration, 2014). We used the DerSimonian and Laird random-effects model to pool the weighted effect of estimates across all trials [9]. The Mantel-Haenszel method was used to estimate study weights. We presented the results using pooled relative risk (RR) for dichotomous outcomes with the corresponding 95% confidence interval (CI). We planned to inspect funnel plots to assess for publication bias if ≥10 trials existed for that outcome [10].

When a trial included more than two arms, each arm was reported in the Table 1. For all main outcomes, only one pair-wise comparison was conducted, and the same groups of participants were only included once in the meta-analysis. For cluster trials, we found the design effect, and then from there calculated the effective sample size which was entered into the primary analyses [8].

**Table 1:** Description of included trials.

Source	Population		Intervention	Control	Primary outcome	Follow-up	Funding
	Demographics	Inclusion Criteria					
Boulware n = 821 United States and Canada Parallel RCT NCT04308668	Median age: 40 yo (IQR 33–50) Women: 51.6% Chronic comorbidities: 27.4% Hypertension: 12.1% Asthma: 7.6% Diabetes: 3.4% Smoker: 3.3% HCW: 66.4% Household contact: 29.8% High risk exposure (no mask or eye shield): 87.6%	“Post-exposure prophylaxis” -≥18yo -Household or occupational exposure to an individual with confirmed COVID-19 -Within 6 feet or less for >10 minutes with the infected individual -May have had no facemask or eye shield OR facemask worn BUT no eye shield	Hydroxychloroquine 800mg PO once, then 600mg PO 6–8 hours later once, then 600mg PO daily for four days for a total course of 5 days	Placebo	Symptomatic illness of COVID-19 and if possible, laboratory confirmed	2 weeks	• -Academic grants • -Part of hydroxychloroquine donated by industry
Rajasingham n = 1483 United States and Canada Parallel RCT NCT04328467	Median age: 41 yo (IQR 34–49) Women: 51.2% Chronic comorbidities: 33.8% Hypertension: 13.8% Asthma: 10.1% Diabetes: 3.4% Smoker: 3.4% HCW: 100% Household contact: 0% High risk exposure (No mask or eye shield): 14.6%	“Pre-exposure prophylaxis” -≥18yo -A healthcare worker at high risk for COVID-19 exposure defined as 1-Working in an emergency department 2-Working in the ICU 3-Working in a COVID-19 hospital ward 3-Performed an aerosol generating procedures 4-First responder	2 intervention arms: 1-Hydroxychloroquine 400mg PO once, followed by 400mg 6 to 8 hours later, then 400 mg PO weekly for 12 weeks 2- Hydroxychloroquine 400mg PO once, followed by 400mg 6 to 8 hours later, then 400 mg PO twice weekly for 12 weeks	Placebo	COVID-19 free survival (defined as symptomatic illness or PCR confirmed)	12 weeks	Academic grant
Mitja n = 2485 Spain Cluster RCT NCT04304053	Mean age: 48.6 yo (SD 19.0) Women: 72.9% Chronic comorbidities: 39.4% Cardiovascular: 13.3% Respiratory: 4.8% Metabolic: 8.4% Smoker: NR HCW: 60.3% Household contact: 27.1% Nursing home residents: 12.7% High risk exposure (No mask or eye shield): 32.8%	“Post-exposure prophylaxis” -≥18yo -Recent history of a close contact to a PCR-confirmed COVID-19 case (i.e. >15minutes within 2 meters, up to 7 days prior to enrollment) -Absence of COVID-19 symptoms within preceding 2 weeks -A healthcare worker, a household contact or a nursing home worker or resident	-Hydroxychloroquine 800mg PO on day 1, then 400mg PO daily for 6 days	Usual care	Symptomatic and PCR proven COVID-19	4 weeks	Crowdfunding and industry funded
Abella n = 132 United States Parallel RCT NCT04329923	Median age: 33 yo (range 20–66) Women: 69% Chronic comorbidities: 29% Hypertension: 21% Asthma: 17% Diabetes: 3% Smoker: 0% HCW: 100% Household contact: 0% High risk exposure (No mask or eye shield): 0%	“Post-exposure prophylaxis” Healthcare workers (physician, nurse, nursing assistant, emergency technicians, respiratory therapists) with practice in the emergency department and dedicated COVID-19 units that 1-Worked ≥20 hours per week in a hospital-based unit 2-Had no history of SARS CoV-2 infection 3-No symptoms suggestive of COVID-19 in the weeks before enrollment	Hydroxychloroquine 600mg PO daily for two months	Placebo	Incidence of SARS CoV-2 infection as determined by a nasopharyngeal swab during 8 weeks of treatment	8 weeks	Philanthropic donations

COVID-19 = Coronavirus disease 2019; HCW = Healthcare workers; ICU = Intensive care unit; IQR = Interquartile range; NR = Not recorded; PCR = Polymerase chain reaction; PO = Per oral; RCT = Randomized clinical trial; SARS CoV-2 = Severe acute respiratory syndrome coronavirus-2; SD = Standard deviation; yo = years old

Statistical heterogeneity was assessed using the Chi^2^ and I^2^ statistics. A Chi^2^  P value of < 0.1 or an I^2^ > 50% qualified as a significant heterogeneity [11]. Heterogeneity between trials was explored by performing pre-defined subgroup analyses to investigate whether certain baseline factors influenced treatment effects. These pre-specified subgroups included: 1-Location of contact with COVID-19 (home versus HCW) (we anticipate less mortality, hospitalization, and rates of COVID-19 in the HCW); 2-Dose of hydroxychloroquine (weekly versus daily) (we anticipate no difference in effect of mortality, hospitalization, or COVID-19 transmission, but more side effects with the daily dosing); and 3-Pre versus post-exposure prophylaxis (we anticipate a greater reduction in hospitalization, mortality and disease transmission in the pre-exposure group, we will not perform subgroup analyses for adverse events using the timing of prophylaxis).

### Sensitivity analysis

Sensitivity analysis was conducted to challenge the robustness of the results and to explore the impact of removing high risk of bias trials and trials that were prepublished. We hypothesized that the treatment effect would be smaller after excluding prepublished trials or trials at high risk of bias. We also performed a sensitivity analysis for cluster RCTs examining the robustness of the effective sample size compared to the unadjusted data set [8].

### Missing data

Four trial authors were contacted for missing or unclear information and one responded. Where email inquiry was not possible or the author did not respond, the available data was analyzed and the potential impact of missing data was reported in the risk of bias section.

### Assessing the certainty of evidence

Two reviewers (KL and DC), independently and in duplicate, used the *Grading of Recommendations Assessment*, *Development and Evaluation* (GRADE) approach to assess the certainty of evidence for each outcome [12]. Reviewers classified the certainty of the evidence as very low, low, moderate, or high using the five GRADE criteria (risk of bias, inconsistency indirectness, imprecision, and publication bias). A very low and low certainty rating indicates that the true effect is probably or may be (respectively) markedly different from the estimated effect [13]. A moderate certainty means that the true effect is probably close to the estimated effect and high certainty indicates there is a lot of confidence that the true effect is similar to the estimated effect [13]. We used the GRADEpro software to prepare the summary of findings (SoF) table [14].

## Results

### Screening

**Fig 1:**
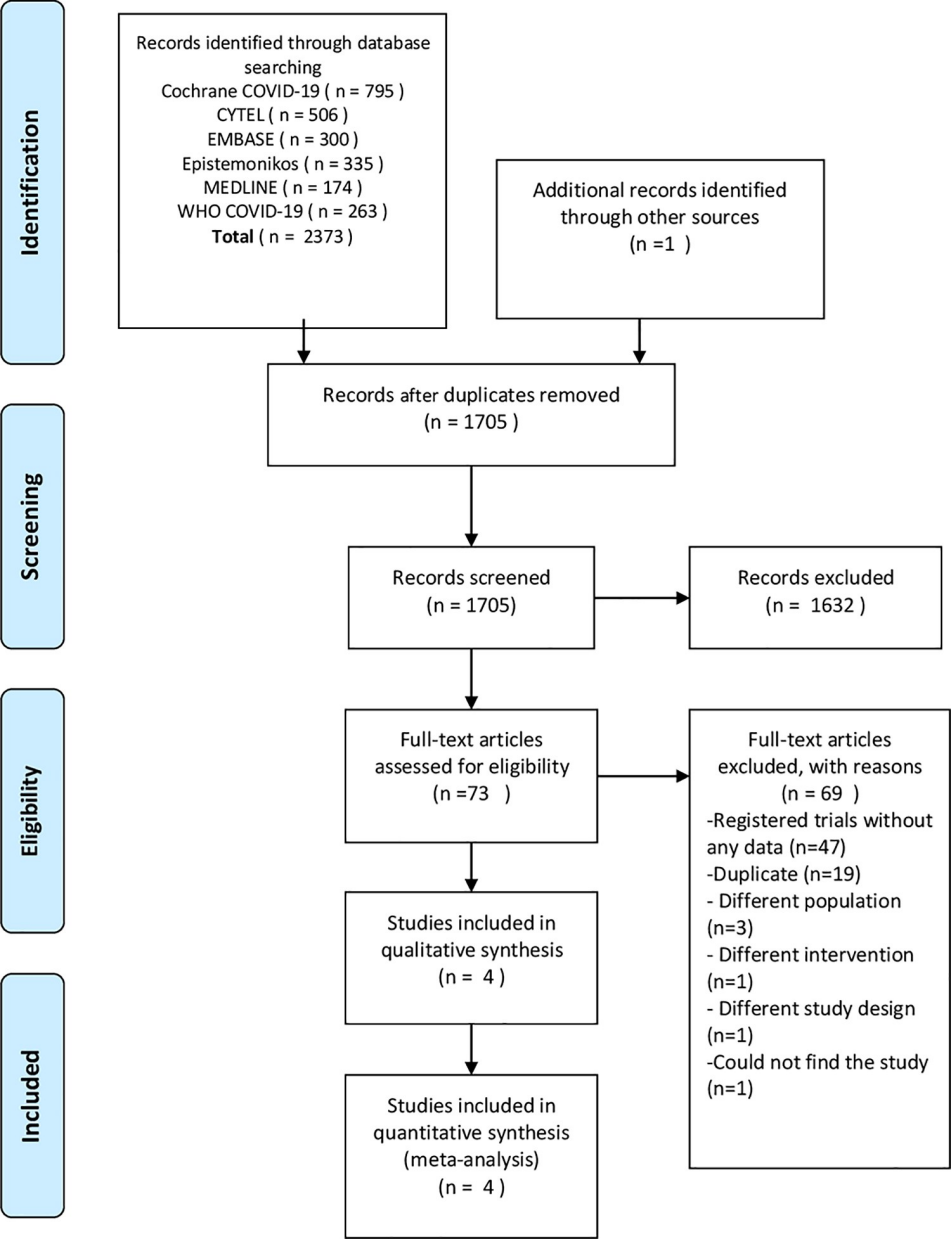
Prisma flow diagram.

The electronic search identified a total of 2374 citations (Fig 1). After removing duplicates, 1705 underwent title and abstract screening, 73 trials remained after exclusion of 1632 citations. Forty-seven of the remaining citations were registered protocols (S5 Table), 22 full text reviews were ineligible (S6 Table), and four trials met the inclusion criteria that underwent a quantitative analysis [15–18]. All trails were peer-reviewed and published in full.

### Characteristics of included trials

Overall, four eligible RCTs enrolling a total of 4921 participants [15–18] were included. Please see Table 1 for a description of included trials. The average age of enrolled participants was 40.7±6.4 years, 61.2% were women, and 32.4% had chronic comorbidities. An average of 81.7% of participants were HCW, and 14.2% were people living with an individual that had a confirmed diagnosis of COVID-19. Only one trial enrolled HCW at high risk for COVID-19 exposure, but did not mandate confirmed COVID-19 contact (i.e. pre-exposure prophylaxis) [17]. The remaining three trials required contact with a COVID-19 patient (i.e. post-exposure prophylaxis) [15, 16, 18]. Thirty four percent of participants had inappropriate PPE donned during their exposures. All trials examined the use of hydroxychloroquine, no trials utilized chloroquine as their intervention. Three trials requested that participants use daily hydroxychloroquine [15, 16, 18] and one trial examined either weekly or twice weekly hydroxychloroquine (please see Table 1 for dosing details) [17]. One trial compared hydroxychloroquine prophylaxis to no prophylaxis [18], while the others compared to placebo [15–17]. The trials prescribed a course of hydroxychloroquine for five days [15], one week [18], eight weeks [16], or 12 weeks [17].

### Risk of bias

Three trials were deemed to have a low risk of bias for all outcomes [15–17] (See S7 Table for a full justification of the ROB assessment). One manuscript was judged to be at high risk of bias for subjective outcomes due to lack of placebo control [18].

### Disease transmission

**Fig 2:**
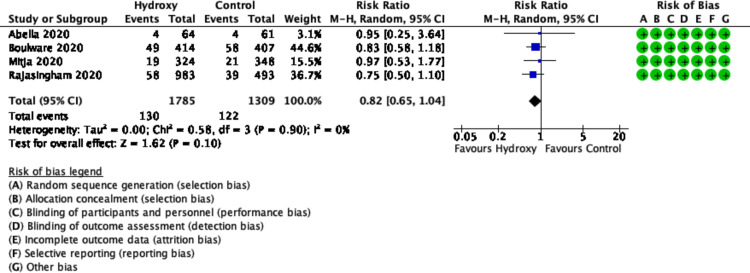
Forest plot of COVID-19 infection.

Four trials enrolling 4921 patients (was reduced to 3094 in the analysis to account for cluster-effect) reported on developing COVID-19 (defined by either symptoms or PCR confirmation) [15–18]. Prophylactic hydroxychloroquine did not reduce the risk of developing COVID-19 (RR 0.82, 95% CI 0.65 to 1.04; I^2^ = 0%, P = 0.90; moderate certainty) (Fig 2) (Table 2 and S8 Table). When only those with positive SARS CoV-2 PCR were analyzed, hydroxychloroquine did not reduce the risk of infection compared to placebo (RR 0.97, 95% CI 0.64 to 1.47; I^2^ = 0%, P = 0.96; moderate certainty) (S1 Fig).

**Table 2:** Abridged summary of findings table.

Outcome	Anticipated absolute effects* (95% CI)		Relative effect (95% CI)	№ of participants (studies)	Certainty of the evidence (GRADE)
	Risk with placebo	Risk with Hydroxychloroquine			
COVID-19 positive	93 per 1,000	**76 per 1,000** (61 to 97)	RR 0.82 (0.65 to 1.04)	3094 (4 RCTs)	⨁⨁⨁◯ MODERATE ^a^
PCR positive	32 per 1,000	**31 per 1,000** (21 to 47)	RR 0.97 (0.64 to 1.47)	3094 (4 RCTs)	⨁⨁⨁◯ MODERATE ^b^
Hospitalizations	10 per 1,000	**7 per 1,000** (3 to 15)	RR 0.72 (0.34 to 1.50)	3094 (4 RCTs)	⨁⨁⨁◯ MODERATE^c^
Mortality	0 per 1,000	**0 per 1,000** (0 to 0)	RR 3.26 (0.13 to 79.74)	3094 (4 RCTs)	⨁⨁◯◯ LOW ^d^
Adverse events	156 per 1,000	**432 per 1,000** (216 to 868)	RR 2.76 (1.38 to 5.55)	2978 (4 RCTs)	⨁⨁⨁◯ MODERATE ^e^
Nausea or dyspepsia	98 per 1,000	**187 per 1,000** (108 to 324)	RR 1.91 (1.10 to 3.31)	2306 (3 RCTs)	⨁⨁⨁◯ MODERATE ^f^
Vomiting or diarrhea	53 per 1,000	**245 per 1,000** (95 to 634)	RR 4.60 (1.78 to 11.91)	2978 (4 RCTs)	⨁⨁⨁◯ MODERATE ^g^
Arrythmia	6 per 1,000	**5 per 1,000** (2 to 11)	RR 0.71 (0.29 to 1.73)	2978 (4 RCTs)	⨁⨁◯◯ LOW ^h^^,^^i^
Vision changes	4 per 1,000	**8 per 1,000** (2 to 26)	RR 2.27 (0.70 to 7.29)	2176 (2 RCTs)	⨁⨁◯◯ LOW ^j^^,^^k^
Compliance	890 per 1,000	**845 per 1,000** (792 to 908)	RR 0.95 (0.89 to 1.02)	1618 (3 RCTs)	⨁⨁◯◯ LOW ^l^^,^^m^

Explanations

a. Rated down for imprecision as there were fewer than 300 events and estimate of effect ranges from 33 fewer to 4 more events.

b. Rated down for imprecision as there were fewer than 300 events and estimate of effect ranged from 12 fewer to 15 more events.

c. Rated down for imprecision as there were fewer than 300 events and estimate of effect ranges from 7 fewer to 5 more events.

d. Rated down for imprecision as there was an extremely small number of total events.

e. Rated down for inconsistency as there was important heterogeneity suggested by an I^2^ = 95%, P<0.00001, df = 3, Chi^2^ = 59.64, different estimates of effect and confidence intervals do not overlap.

f. Rated down for inconsistency as there was important heterogeneity suggested by an I^2^ = 74%, P = 0.02, df = 2, Chi^2^ = 7.63, different estimates of effect and confidence intervals do not overlap.

g. Rated down for inconsistency as there was important heterogeneity suggested by an I^2^ = 92%, P<0.00001, df = 3, Chi^2^ = 36.56, different estimates of effect and confidence intervals do not overlap.

h. Rated down for indirectness as the outcome of arrhythmia varies in patient importance from atrial fibrillation to ventricular tachycardia.

i. Rated down for imprecision as there were fewer than 300 events and estimate of effect ranges from 5 fewer to 5 more events.

j. Rated down for indirectness as visual changes vary in patient importance from mild dizziness to blindness.

k. Rated down for imprecision as there were fewer than 300 events and estimate of effect ranges from 1 fewer to 22 more events.

l. Rated down for inconsistency as there was important heterogeneity suggested by an I^2^ = 61%, P = 0.08, Chi^2^ = 5.10, df = 2, and some confidence intervals do not overlap.

m. Rated down for imprecision as even though there was greater than 300 events, the estimate of effect ranges from 98 fewer to 18 more events.

### Hospitalizations

**Fig 3:**
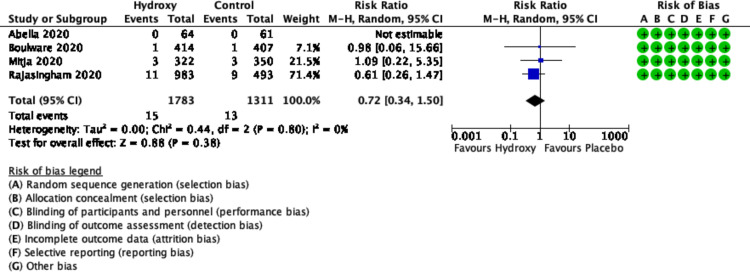
Forest plot of hospitalizations.

All four trials (n = 3094 participants) reported on hospitalization [15–18]. The use of hydroxychloroquine compared to placebo did not reduce the risk of hospitalizations (RR 0.72, 95% CI 0.34 to 1.50; I^2^ = 0%, P = 0.80; moderate certainty) (Fig 3).

### Mortality

**Fig 4:**
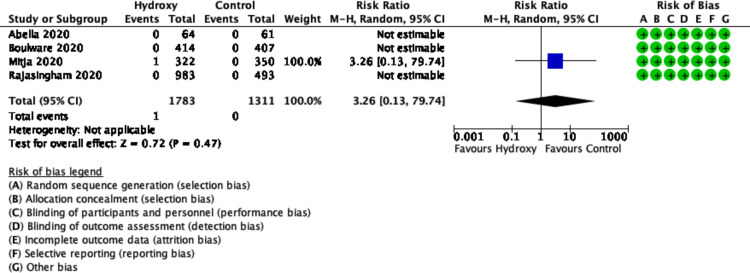
Forest plot of mortality.

The pooled results from four trials (n = 3094 participants) showed uncertain effect of hydroxychloroquine on mortality (RR 3.26, 95% CI 0.13 to 79.74; heterogeneity not applicable; low certainty) (Fig 4) [15–18].

### Adverse events

**Fig 5:**
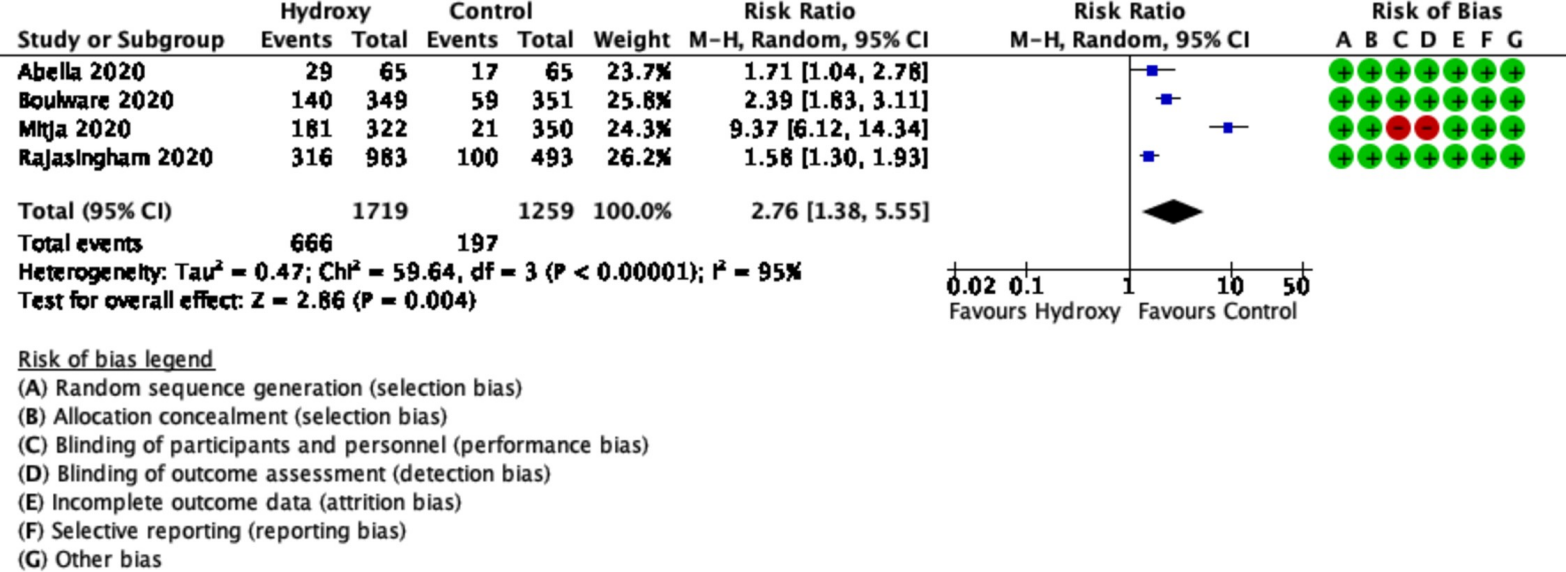
Forest plot of ≥1 adverse event.

The pooled result from four trials (n = 2978 participants) found hydroxychloroquine increased the risk of adverse events (RR 2.76, 95% CI 1.38 to 5.55; I^2^ = 95%, P<0.00001; moderate certainty) (Fig 5) [15–18].

### Gastrointestinal adverse events

The pooled estimate from three trials (n = 2306 participants) showed increased the risk of nausea and dyspepsia with hydroxychloroquine (RR 1.91, 95% CI 1.10 to 3.31; I^2^ = 74%, P = 0.02; moderate certainty) (S2 Fig) [15–17]. Four trials (n = 2978 participants) reported on vomiting or diarrhea and found increased risk with hydroxychloroquine use (RR 4.60, 95% CI 1.78 to 11.91; I^2^ = 92%, P<0.00001; moderate certainty) (S3 Fig) [15–18].

### Arrhythmia

**Fig 6:**
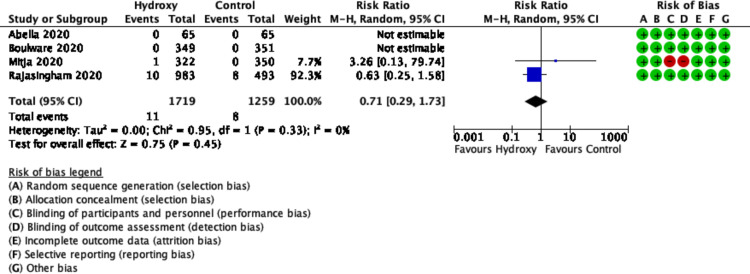
Forest plot of arrhythmia.

Data on arrhythmias was available from four trials (n = 2978 participants) [15–18]. The effect of hydroxychloroquine on the risk of arrhythmias, compared placebo, was uncertain (RR 0.71, 95% CI 0.29 to 1.73; I^2^ = 0%, P = 0.33; low certainty) (Fig 6).

### Visual changes

Only two trials (n = 2176 participants) reported on the risk of developing visual changes [15, 17]. The pooled estimate showed uncertain effect of hydroxychloroquine on visual changes compared to placebo (RR 2.27, 95% CI 0.70 to 7.29; I^2^ = 0%, P = 0.41; low certainty) (S4 Fig).

### Compliance

Three trials (n = 1618) examined compliance [15, 16, 18]. The pooled estimate showed no difference in compliance to hydroxychloroquine versus placebo (RR 0.95, 95% CI 0.89 to 1.02; I^2^ = 61%, P = 0.08; low certainty) (S5 Fig).

### Subgroup analysis

Subgroup analysis suggested a higher risk of vomiting and diarrhea with daily dosing compared to weekly dosing (P-interaction = 0.04), but no subgroup effect was seen for any other outcomes (S9 Table). We were unable to complete the subgroup analysis for location of COVID-19 contact (home versus HCW).

### Sensitivity analysis

We planned three sensitivity analyses for all outcomes. Sensitivity analyses excluding high risk of bias trials or using the full sample size did not alter the results for most outcomes. The only notable change was when high risk of bias studies were excluded, compliance was statistically better in those that took placebo compared to active drug. We were unable to perform sensitivity analyses for the outcomes of mortality, nausea/dyspepsia, or vision changes (S10 Table). In addition, all trials were published in a peer review journal, therefore we did not perform any subgroup analyses by publication status.

## Discussion

In this systematic review and meta-analysis, we included four RCTs (n = 4921 participants) of both pre- and post-exposure prophylaxis with hydroxychloroquine compared to placebo or no prophylaxis. Overall, we demonstrated with moderate certainty of evidence that prophylaxis with hydroxychloroquine increases adverse events without reducing the risks of developing COVID-19 (moderate certainty), hospitalization (moderate certainty), or mortality (low certainty).

Both chloroquine and hydroxychloroquine have *in vitro* activity against a variety of viruses including SARS CoV-2 and SARS CoV-1 [19–23]. One possible mechanism is by impairing the terminal glycosylation of the angiotensin-converting-enzyme 2 (ACE2) receptor, blocking the binding site for the envelope spike glycoprotein and therefore inhibiting propagation of the virus in the human body [24]. However, hydroxychloroquine was found to have superior *in vitro* antiviral activity compared to chloroquine [25] and hydroxychloroquine is less likely to accumulate in tissue and thus avoids serious adverse events such as retinopathy and cardiomyopathy [26, 27]. Hydroxychloroquine was therefore quickly identified as a potential solution to defeat SARS CoV-2 [28]. Although some small observational studies demonstrated antiviral benefit of hydroxychloroquine in patients infected with SARS CoV-2 [29, 30], the Randomise Evaluation of COVid-19 thERapy (RECOVERY) RCT proved different [31, 32]. This trial randomized hospitalized patients to either receive hydroxychloroquine (n = 1542) or usual care (n = 3132). They found there was no difference in the primary endpoint of 28-day mortality (Hazard ratio 1.11, 95% CI 0.98–1.26, p = 0.10) [31, 32]. There was also no beneficial effect on hospital length of stay [31, 32]. Similarly, the SOLIDARITY trial discontinued the trial’s hydroxychloroquine arm citing no difference in mortality compared to usual care [33]. Originally, the lack of effect was thought to be secondary to enrollment of patients with severe infections, however a recently published RCT of patients with mild to moderate COVID-19 found that hydroxychloroquine alone or with azithromycin did not improve overall clinical status of patients at day 15 post randomization [34].

One major criticism of treatment trials is perhaps therapy is being initiated too late after the viral infection. In addition, trials of treatments administered in hospital were doing little to break the chain of transmission, hence was born the suggestion of prophylaxis. After a large COVID-19 exposure in a long-term care hospital, 211 participants, including 189 patients and 22 HCWs were given hydroxychloroquine 400mg PO daily for a total of 14 days. All PCR tests after the 14 days of treatment were negative. The prophylaxis was completed by 96.5% of participants, with the most common side effect being diarrhea [35]. Despite these results, recent large RCTs have all failed to demonstrate a reduction in transmission of SARS CoV-2 with the administration of hydroxychloroquine [15–18]. However, there is considerable variation in duration of treatment and follow up. It is possible that treatment and follow up were not long enough to capture a sufficient number of events, particularly as the incidence of active COVID-19 decreased as the first wave was controlled.

There was high heterogeneity for the outcomes of adverse events, nausea, and vomiting and diarrhea. This is unlikely to be explained by different populations, as the baseline characteristics reported between trials were fairly similar. We did attempt to explain these through a subgroup analyses, particularly with daily versus weekly dosing. Although the lack of subgroup effect based on dosing was surprising as the elimination half-life of hydroxychloroquine is long (5–40 days) [36], allowing for more frequent dosing to cause higher levels and drug accumulation, however this subgroup analysis was likely underpowered [37] and more data may demonstrate a dose-response effect. Another potential explanation of the heterogeneity is the difference in daily dosing regimens. For instance, daily dosing ranged from 400mg to 600mg daily, and for a duration of time from five days to two months, albeit this is speculative.

To our knowledge, this is the first systematic review on this novel topic. There are several strengths of this review, we adhered to a rigorous process with an extensive systematic search of the literature, summary of current trial registries, duplication of all aspects of the review, and adherence to PRISMA guidelines (S11 Table) [38].

However, our report has some limitations. First, we were unable to complete some of our pre-specified outcomes of severity of COVID-19 symptoms, duration of COVID-19 symptoms, and admission to the ICU. This is due to the fact that there is little primary research on this topic and lack of patient-level data. Therefore, as the trials which we summarized (S5 Table) complete and publish their findings, we may be able to assess the effect of hydroxychloroquine on disease severity. The lack of patient data limited our subgroup analyses. We were unable to assess if there was effect modification by location of contact with COVID-19. Second, we were unable to examine funnel plots to detect publication bias given the small number of trials for each outcome. Nevertheless, we attempted to reduce publication bias by implementing a comprehensive search strategy. In addition, given the rapidity of the systematic review, we did not register or publish our protocol, although all outcomes were defined *a priori*. Another limitation was the available data. Given the evolving knowledge in this area, a repeated systematic review may be required to gain certainty in the evidence [39].

## Conclusion

In conclusion, low certainty evidence showed that COVID-19 pharmacologic prophylaxis with hydroxychloroquine is ineffective at improving patients’ outcomes and is associated with higher risk of adverse events.

## Supporting information

S1 Fig Forest plot of SARS CoV-2 PCR positive. (DOCX)

S2 Fig Forest plot of nausea and dyspepsia. (DOCX)

S3 Fig Forest plot of vomiting or diarrhea. (DOCX)

S4 Fig Forest plot of visual changes. (DOCX)

S5 Fig Forest plot of compliance with the medication. (DOCX)

S1 Table Search strategy of Embase. (DOCX)

S2 Table Search strategy for Cochrane COVID-19 register of controlled trials. (DOCX)

S3 Table Search strategy of Epistemonikos. (DOCX)

S4 Table Search strategy of World Health Organization (WHO) International Clinical Trials Registry Platform (ICTRP). (DOCX)

S5 Table Description of eligible registered clinical trials with no extractable data. (DOCX)

S6 Table Ineligible articles. (DOCX)

S7 Table Risk of bias justifications. (DOCX)

S8 Table Summary of findings table. (DOCX)

S9 Table Summary table of subgroup analyses. (DOCX)

S10 Table Summary table of sensitivity analyses. (DOCX)

S11 Table PRISMA checkbox. (DOCX)
