# Supplementary figures and images for: The efficacy and safety of hydroxychloroquine for COVID-19 prophylaxis: A systematic review and meta-analysis of randomized trials
Source: PLoS One. 2021 Jan 6;16(1):e0244778. doi: 10.1371/journal.pone.0244778 (PMC7787432; doi:10.1371/journal.pone.0244778)

S1 Fig: Forest plot of SARS-CoV-2 PCR positive


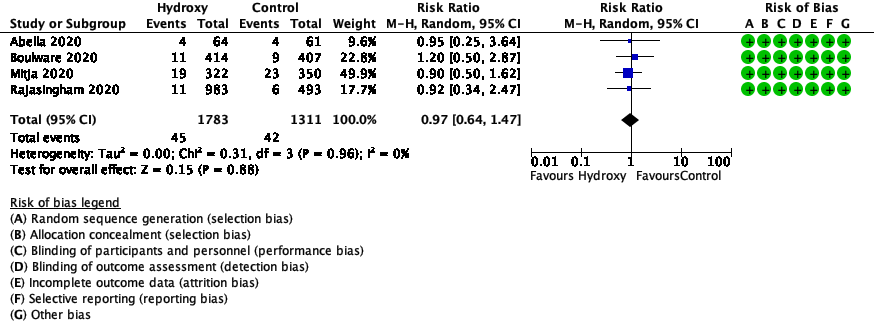

Supplement: S1 Fig — (DOCX) [file pone.0244778.s001.docx]

S2 Fig: Forest plot of nausea and dyspepsia


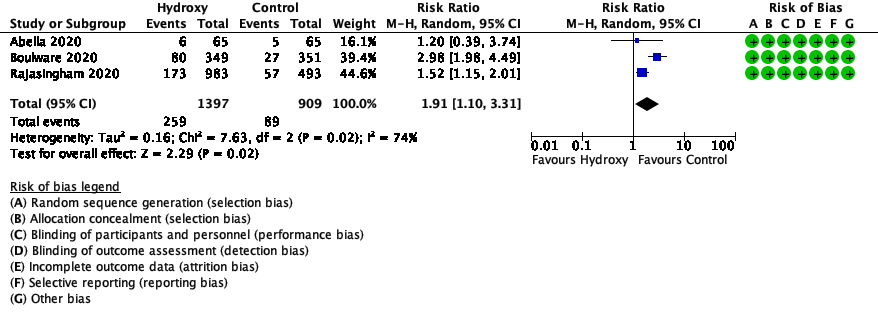

Supplement: S2 Fig — (DOCX) [file pone.0244778.s002.docx]

S3 Fig: Forest plot of vomiting or diarrhea


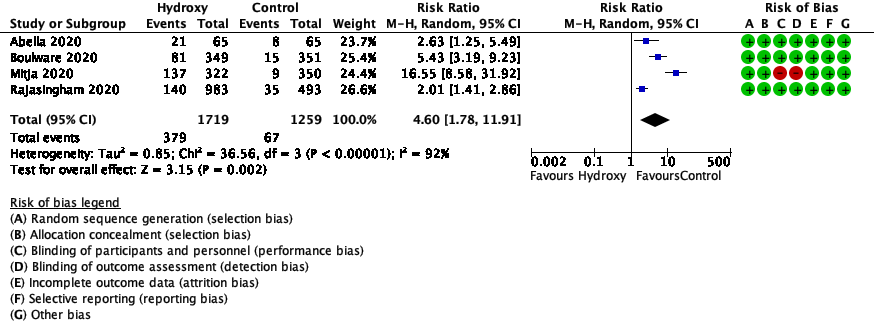

Supplement: S3 Fig — (DOCX) [file pone.0244778.s003.docx]

S4 Fig: Forest plot of visual changes


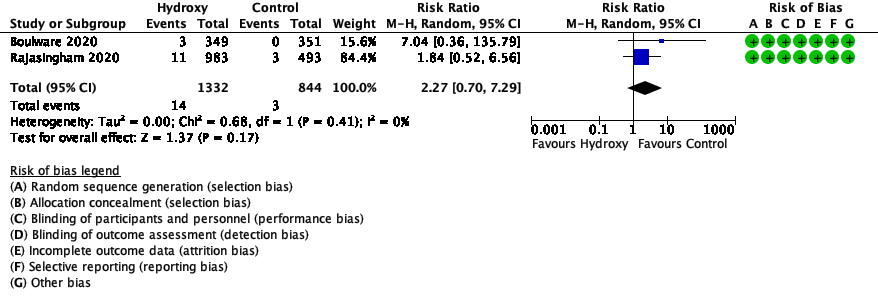

Supplement: S4 Fig — (DOCX) [file pone.0244778.s004.docx]

S5 Fig: Forest plot of compliance with the medication


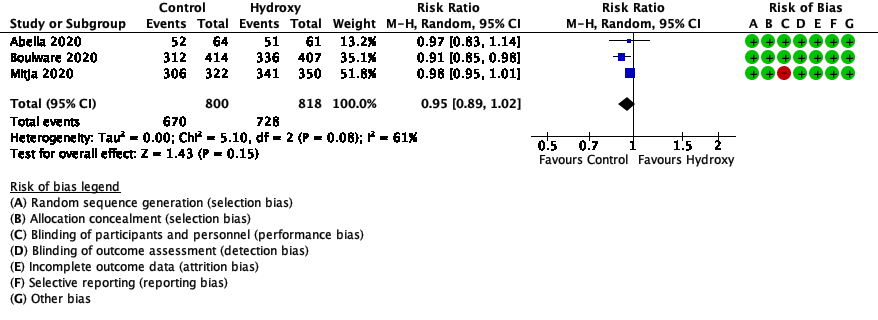

Supplement: S5 Fig — (DOCX) [file pone.0244778.s005.docx]
