# Supplementary material for: The efficacy and safety of hydroxychloroquine for COVID-19 prophylaxis: A systematic review and meta-analysis of randomized trials
Source: PLoS One. 2021 Jan 6;16(1):e0244778. doi: 10.1371/journal.pone.0244778 (PMC7787432; doi:10.1371/journal.pone.0244778)
Supplement: S1 Table — (DOCX) [file pone.0244778.s006.docx]

S1 Table: Search Strategy of Embase

**Embase <1974 to 2019 August 10>, and OVID Medline Epub Ahead of Print, In-Process & Other Non-Indexed Citations, Ovid MEDLINE(R) Daily and Ovid MEDLINE(R) 1946 to Present**

1 exp chloroquine/ or exp hydroxychloroquine/ (75541)

2 chloroquine.mp. or hydroxychloroquine.ti,ab,kw. [mp=ti, ab, hw, tn, ot, dm, mf, dv, kw, fx, dq, nm, kf, ox, px, rx, ui, sy] (73993)

3 (886u3h6uff or aralen or arechine or arequin or chingamin or chlorochin or chloroquine or khingamin or nivaquine or oe48649k6n or hydroxychlorochin* or oxychlorochin or oxychloroquine).mp. or plaquenil.ti,ab,kw. [mp=ti, ab, hw, tn, ot, dm, mf, dv, kw, fx, dq, nm, kf, ox, px, rx, ui, sy] (62682)

4 1 or 2 or 3 (89771)

5 exp Coronaviridae/ (44705)

6 exp Coronaviridae Infections/ (45273)

7 coronavirus*.mp. or (COVID or "covid-19" or COVID19 or nCoV or hCoV or 19nCoV or 2019nCoV or 2019-nCoV or SARS-CoV-2 or SARS-CoV or SARSCOV2 or SARSCOV-2 or SARS2 or MERS-CoV).ti,ab,kw. (115152)

8 (coronavirus* or corona virus* or betacoronavirus* or OC43 or NL63 or 229E or HKU1 or HCoV* or ncov* or covid* or sars-cov* or sarscov* or Sars-coronavirus* or Severe Acute Respiratory Syndrome Coronavirus*).mp. (122639)

9 or/5-8 (135546)

10 9 not (SARS or SARS-CoV or MERS or MERS-CoV or Middle East respiratory syndrome or camel* or dromedar* or equine or coronary or coronal or covidence* or covidien or influenza virus or HIV or bovine or calves or TGEV or feline or porcine or BCoV or PED or PEDV or PDCoV or FIPV or FCoV or SADS-CoV or canine or CCov or zoonotic or avian influenza or H1N1 or H5N1 or H5N6 or IBV or murine corona*).mp. (68850)

11 ((pneumonia or covid* or coronavirus* or corona virus* or ncov* or 2019-ncov or sars*).mp. or exp pneumonia/) and (Wuhan or Hubei or China or Chinese).mp. (22917)

12 (2019-ncov or ncov19 or ncov-19 or 2019-novel CoV or 19nCoV or sars-cov2 or sars-cov-2 or sarscov2 or sarscov-2 or Sars-coronavirus2 or Sars-coronavirus-2 or SARS-like coronavirus* or coronavirus-19 or covid19 or covid-19 or covid 2019 or ((novel or new or nouveau or "19" or "2019") adj2 (CoV or nCoV or covid or coronavirus* or corona virus or Pandemi*2)) or ((covid or covid19 or covid-19) and pandemic*2) or (coronavirus* and pneumonia)).mp. (87944)

13 COVID-19.rx,px,ox. or severe acute respiratory syndrome coronavirus 2.os. (16650)

14 COVID-19.mp. or severe acute respiratory syndrome coronavirus 2.dj,dq. [mp=ti, ab, hw, tn, ot, dm, mf, dv, kw, fx, dq, nm, kf, ox, px, rx, ui, sy] (74443)

15 or/10-14 (112290)

16 4 and 15 (3442)

17 (Randomized Controlled Trial or Controlled Clinical Trial or Pragmatic Clinical Trial or Equivalence Trial or Clinical Trial, Phase III).pt. (603321)

18 Randomized Controlled Trial/ (1126001)

19 exp Randomized Controlled Trials as Topic/ (322195)

20 "Randomized Controlled Trial (topic)"/ (183862)

21 Controlled Clinical Trial/ (557955)

22 exp Controlled Clinical Trials as Topic/ (334948)

23 "Controlled Clinical Trial (topic)"/ (10927)

24 Randomization/ (190912)

25 Random Allocation/ (187079)

26 Double-Blind Method/ (309181)

27 Double Blind Procedure/ (174838)

28 Double-Blind Studies/ (291869)

29 Single-Blind Method/ (66638)

30 Single Blind Procedure/ (39806)

31 Single-Blind Studies/ (68695)

32 Placebos/ (331611)

33 Placebo/ (353260)

34 Control Groups/ (112155)

35 Control Group/ (112155)

36 (random* or sham or placebo*).ti,ab,hw,kf,kw. (3580088)

37 ((singl* or doubl*) adj (blind* or dumm* or mask*)).ti,ab,hw,kf,kw. (546324)

38 ((tripl* or trebl*) adj (blind* or dumm* or mask*)).ti,ab,hw,kf,kw. (2410)

39 (control* adj3 (study or studies or trial* or group*)).ti,ab,kf,kw. (2375759)

40 (Nonrandom* or non random* or non-random* or quasi-random* or quasirandom*).ti,ab,hw,kf,kw. (100321)

41 allocated.ti,ab,hw. (152134)

42 ((open label or open-label) adj5 (study or studies or trial*)).ti,ab,hw,kf,kw. (99046)

43 ((equivalence or superiority or non-inferiority or noninferiority) adj3 (study or studies or trial*)).ti,ab,hw,kf,kw. (20975)

44 (pragmatic study or pragmatic studies).ti,ab,hw,kf,kw. (1015)

45 ((pragmatic or practical) adj3 trial*).ti,ab,hw,kf,kw. (10740)

46 ((quasiexperimental or quasi-experimental) adj3 (study or studies or trial*)).ti,ab,hw,kf,kw. (20683)

47 (phase adj3 (III or "3") adj3 (study or studies or trial*)).ti,hw,kf,kw. (120448)

48 or/17-47 (5256815)

49 16 and 48 (474)

50 49 use ppez (174)

51 49 use oemezd (300)

52 remove duplicates from 49 (316)
