# Supplementary material for: The efficacy and safety of hydroxychloroquine for COVID-19 prophylaxis: A systematic review and meta-analysis of randomized trials
Source: PLoS One. 2021 Jan 6;16(1):e0244778. doi: 10.1371/journal.pone.0244778 (PMC7787432; doi:10.1371/journal.pone.0244778)
Supplement: S2 Table — (DOCX) [file pone.0244778.s007.docx]

S2 Table: Search strategy for Cochrane COVID-19 Register of Controlled Trials

**COCHRANE COVID-19**(<https://covid-19.cochrane.org/>)

**Search 1:** chloroquine OR hydroxychloroquine OR 886u3h6uff or aralen or arechine or arequin or chingamin or chlorochin or khingamin or nivaquine or oe48649k6n or hydroxychlorochin* or oxychlorochin or oxychloroquine or plaquenil
