# Supplementary material for: The efficacy and safety of hydroxychloroquine for COVID-19 prophylaxis: A systematic review and meta-analysis of randomized trials
Source: PLoS One. 2021 Jan 6;16(1):e0244778. doi: 10.1371/journal.pone.0244778 (PMC7787432; doi:10.1371/journal.pone.0244778)
Supplement: S3 Table — (DOCX) [file pone.0244778.s008.docx]

S3 Table: Search strategy of Epistemonikos

**Epistemonikos COVID-19 Evidence**

“prevention or treatment” and (Hydroxychloroquine or Chloroquine), Filtered primary studies for “RCT”
