# Supplementary material for: The efficacy and safety of hydroxychloroquine for COVID-19 prophylaxis: A systematic review and meta-analysis of randomized trials
Source: PLoS One. 2021 Jan 6;16(1):e0244778. doi: 10.1371/journal.pone.0244778 (PMC7787432; doi:10.1371/journal.pone.0244778)
Supplement: S4 Table — (DOCX) [file pone.0244778.s009.docx]

S4 Table: Search Strategy of World Health Organization (WHO) International Clinical Trials Registry Platform (ICTRP)

**WHO COVID-19 Database** (20 July 2020) **(**[**https://www.who.int/emergencies/diseases/novel-coronavirus-2019/global-research-on-novel-coronavirus-2019-ncov**](https://www.who.int/emergencies/diseases/novel-coronavirus-2019/global-research-on-novel-coronavirus-2019-ncov)**)**

Title, abstract, subject: chloroquine OR hydroxychloroquine OR 886u3h6uff or aralen or arechine or arequin or chingamin or chlorochin or khingamin or nivaquine or oe48649k6n or hydroxychlorochin* or oxychlorochin or oxychloroquine or plaquenil

Filtered to exclude Medline database

Results: 263 citations
