# Supplementary material for: The efficacy and safety of hydroxychloroquine for COVID-19 prophylaxis: A systematic review and meta-analysis of randomized trials
Source: PLoS One. 2021 Jan 6;16(1):e0244778. doi: 10.1371/journal.pone.0244778 (PMC7787432; doi:10.1371/journal.pone.0244778)
Supplement: S5 Table — (DOCX) [file pone.0244778.s010.docx]

S5 Table: Description of eligible registered clinical trials with no extractable data

| Trial Name/Acronym/PI | Registration Number | Inclusion Criteria | Intervention | Control | Status | Sponsor |
| --- | --- | --- | --- | --- | --- | --- |
| Post Exposure Prophylaxis in Healthcare Workers Exposed to COVID-19 Patients^1^/HCQ-COVID19/Nasser Al Ansari | NCT04437693 | 18 years or older, all healthcare workers in direct contact with COVID-19 patients | Hydroxychloroquine 400mg twice a day on day 1 followed by 400 mg weekly for 7 weeks. | Placebo | Not yet recruiting | Hamad Medical Corporation |
| Does Hydroxychloroquine Before & During Patient Exposure Protect Healthcare Workers From Coronavirus?^2^/HEROs/Megan Landes | NCT04374942 | Age ≥18 years, health care worker (HCW) in the emergency department who is anticipated to work at least 10 shifts over the duration of the study period (minimum 6 hours per shift) and anticipated to remain in the emergency department for the duration of the study | Oral Hydroxychloroquine, 400 mg taken once daily, for three months as pre-exposure prophylaxis to prevent COVID-19 in health care workers in the emergency department. | Placebo | Enrolling by invitation | Megan Landes |
| Military COVID-19 Hydroxychloroquine Pre-exposure and Post-exposure Prophylaxis Study^3^/Angela Phillips | NCT04343677 | All mission critical personnel enrolled to the DiLorenzo Tricare Health Clinic or Pentagon Flight Medicine Clinic unable to telework or appropriately socially distance with access to the Pentagon during the declared public health crisis | Low dose prophylaxis treated with 200mg Hydroxychloroquine daily  High dose prophylaxis treated with 400mg Hydroxychloroquine daily  Post-exposure arm treated with 400mg Hydroxychloroquine daily for 7 days | Placebo | No longer active | United States Department of Defense |
| Hydroxychloroquine as Post Exposure Prophylaxis for SARS-CoV-2^5^/HOPE Trial/Yong Goo Song | NCT04330144 | Contact person from confirmed case of SARS-CoV-2 infection, medical staff exposed from confirmed case of SARS-CoV-2 infection in hospitals, or persons exposed to SARS-CoV-2 in COVID-19 outbreak situation with certain workplaces, religious groups, and military, etc. | Hydroxychloroquine 800mg daily po 2-5dy: Hydroxychloroquine 400mg daily po | No treatment | Not yet recruiting | Gangnam Severance Hospital |
| Hydroxychloroquine Post-Exposure Prophylaxis for Coronavirus Disease (COVID-19) Among Health-Care Workers^6^/Nesrin Ghanem-Zoubi | NCT04438837 | Age >18 years, HCW who had a contact without adequate personal protective equipment (PPE) with a confirmed COVID-19 patient.Time between exposure to randomization no longer than 72 hours. | Hydroxychloroquine 400mg BID in the first day followed by 200mg BID for overall 10 days | No intervention | Not yet recruiting | Rambam Health Care Campus |
| Prevention of SARS-CoV-2 in Hospital Workers s Exposed to the Virus^7^ /PREP-COVID/Jean Ma Treluyer | NCT04344379 | Age over 18, hospital workers working at AP-HP hospitals, no signs of COVID-19 infection | Hydroxychloroquine 200 mg BID & Azithromycin 250mg daily | Placebo | Suspended | Assistance Publique - Hôpitaux de Paris |
| Hydroxychloroquine in the Prevention of COVID-19 Infection in Healthcare Workers^8^/Peter A McCullough | NCT04333225 | Healthcare workers ≥ 18 to ≤ 75 years with one day or more of exposure to suspect and/or positive COVID-19 patients, including but not limited to those working in the Emergency Department or Intensive Care Unit or unprotected exposure to a known positive COVID-19 patient within 72 hours of screening | Oral hydroxychloroquine 400 mg twice a day on day 1 followed by two 200 mg tablets once a week for a total of 7 weeks | No Intervention | Active, not recruiting | Baylor Research Institute |
| Pre-Exposure Prophylaxis with Hydroxychloroquine for High-Risk Healthcare Workers during the COVID-19 Pandemic ^9^/PrEP_COVID/Jose Muñoz Gutiérrez | NCT04331834 | Healthcare worker age ≥ 18 years, negative PCR and negative serology on day 0 | Hydroxychloroquine 400 mg daily during the first 4 days, followed by 400 mg weekly during 6 months | Placebo | Suspended | Barcelona Institute for Global Health |
| Hydroxychloroquine for Post-Exposure Prophylaxis of COVID-19 among naval personnel: a placebo-controlled, randomized, clinical trial^10^ | U1111-1251-3613 | Naval personnel age 18-59.9, exposure to a patient with confirmed COVID-19 infection (rRT-PCR positive for SARS-CoV-2 virus). Exposure should be for >30 mins within 2 meters of the infected individual and should be within ONE WEEK at the time of inclusion in the study | Loading dose of oral hydroxycholoquine 400mg 12 hourly Day 1 followed by oral HCQ 200mg 12 hourly for the next 4 days. | Placebo |  |  |
| Hydroxy Chloroquine, in open labelled, Randomised intervention for prevention of new infection and adverse outcomes following COVID-19 infection- A Tertiary Hospital based study^12^/Remesh Bhasi | CTRI/2020/03/024402 | Age 18 to 80, moderate to high risk of exposure to infected patients, healthy at the time of enrolment without any symptoms suggestive of any viral infection | Hydroxychloroquine 400 mg bd for one day followed by 400 mg weekly for 7 weeks  Hydroxychloroquine 300 mg daily x 7 days followed by 300 mg weekly x 7 weeks | Not specified | Not yet recruiting | Aster Malabar Institute of Medical Sciences |
| Efficacy of Hydroxychloroquine (HCQ) as Post Exposure Prophylaxis (PEP) for Prevention of COVID-19^13^/PEP-CQ/Deba P Dhibar | NCT04408456 | Asymptomatic individuals who have undertaken International travel in last 2 weeks, or asymptomatic individual with direct contact with laboratory confirmed cases | Hydroxychloroquine 400 mg q 12 hourly on day one followed by 400 mg once weekly for 3 weeks (total cumulative dose of 2000 mg) | No treatment | Comepleted | Postgraduate Institute of Medical Education and Research |
| Hydroxychloroquine Efficacy and Safety in Preventing SARS-CoV-2 Infection and COVID-19 Disease Severity During Pregnancy^14^/COVID-Preg/Raquel González | NCT04410562 | Presenting with fever (≥37.5ºC) and/or one mild symptom suggestive of COVID-19 disease (cough, dyspnoea, chills, odynophagia, diarrhoea, muscle pain, anosmia, taste disorder, headache) OR contact of a SARS-CoV-2 confirmed or suspected case in the past 14 days | Hydroxychloroquine 400 mg/day for three days, followed by 200 mg/day for 11 days | Placebo | Recruiting | Barcelona Institute for Global Health |
| PRECOV: a randomized controlled clinical trial on the effects of hydroxychloroquine in the prevention of COVID-19 in healthcare workers at risk^15^/PRECOV | 2020-001987-28 | Age 18 to 70 years, RT-PCR negative for SARS-CoV-2, antibody test for negative SARS-CoV-2 | Hydroxychloroquine 200mg po | Not specified | Ongoing | OSPEDALE SAN RAFFAELE |
| Hydroxychloroquine to Prevent COVID-19 Disease Amongst Healthcare Workers^16^/PROVIDE/Kimberley Lewis | NCT04371523 | - Healthcare workers age 18 or older, with primary practice in intensive care unit, general internal medicine, - COVID-19 testing centres, emergency rooms, and nursing homes. COVID_19 symptom free at the time of randomization and have a negative diagnostic swab | Hydroxychloroquine sulfate 400mg PO BID on day one, then 400mg PO weekly for two months total. | Placebo | Not yet recruiting | St. Joseph's Healthcare Hamilton |
| Healthcare Worker Exposure Response and Outcomes of Hydroxychloroquine^17^/HERO-HCQ/Adrian Hernandez, MD | NCT04334148 | Age ≥ 18 years old, currently working in any environment in which there is a risk of exposure to patients with COVID-19 infections ("healthcare worker") | Hydroxychloroquine 600mg bid loading dose on day 1 followed by 400mg on days 2-30. | Placebo | Recruiting | Adrian Hernandez |
| Assessment of the Efficacy and Safety of (HCQ) as a Prophylaxis for COVID19 for Health Professionals^18^/COVID_2Pro/Jalila Ben Khelil, Pr | NCT04349228 | - Age 18 to 65, diagnosis of COVID-19 negative, work in a medical intensive care unit exposed to COVID-19 infection | Hydroxychloroquine 200mg/day | Placebo | Withdrawn | Abderrahmane Mami Hospital |
| Hydroxychloroquine Chemoprophylaxis in Healthcare Personnel in Contact With COVID-19 Patients^19^ /PHYDRA/  Jorge Rojas-Serrano | NCT04318015 | 18 years or older, healthcare personnel exposed to patients with COVID-19 respiratory disease | Hydroxychloroquine 200mg per day for 60 days | Placebo | Recruiting | National Institute of Respiratory Diseases, Mexico (Sanofi collaborator) |
| Protect: Study With Hydroxychloroquine for Prevention and Early Phase Treatment of Coronavirus Disease (COVID-19)^20^/PROTECT/Giovanni Martinelli | NCT04363827 | Aged >= 18 years, SARS-CoV-2-exposed subjects, as household members and/or contacts of COVID-19 patients (Group 1). In this group are included Health care professionals in contact with COVID-19 patients, absence of any COVID-19 symptom in last week before randomization (fever >37.5°C, cough, dyspnea) (only for group 1 subjects) | A loading dose Hydroxychloroquine 400 mg twice daily at day 1, followed by a weekly dose of Hydroxychloroquine 200 mg twice daily on days 8, 15 and 22, covering a total of 1 month of treatment. | Observation | Recruiting | Istituto Scientifico Romagnolo per lo Studio e la cura dei Tumori |
| Hydroxychloroquine for COVID-19 Post-exposure Prophylaxis^21^/PEP/Ruanne V. Barnabas | NCT04328961 | - Age 18 to 80 inclusive, had a close contact of a person (index) with known PCR-confirmed SARS-CoV-2 infection or who is currently being assessed for COVID-19. Close contact defined as:   1. Household contact (i.e., residing with the index case in the 14 days prior to index diagnosis)   2. Medical staff, first responders, or other care persons who cared for the index case without personal protection (mask and gloves) - Less than 4 days since last exposure (close contact with a person with SARS-CoV-2 infection) to the index case - Body weight < 100 kg (self-reported) - Access to device and internet for Telehealth visits | Hydrochloroquine 400 mg orally daily for 3 days, then 200 mg orally daily for an additional 11 days | Ascorbic acid 500 mg orally daily for 3 days, then 250 mg orally daily for 11 days | Active, not recruiting | University of Washington |
| chemoPROphyLaxIs with hydroxychloroquine For covId-19 infeCtious disease (PROLIFIC) to prevent covid-19 infection in frontline healthcare workers: a structured summary of a study protocol for a randomised controlled trial^22^/PROLIFIC/Joseph Cheriyan | NCT04352933 | Aged 18 years to 70 years, not previously have been diagnosed with COVID-19, work in a high-risk secondary or tertiary healthcare setting (hospitals accepting COVID-19 patients) with direct patient-facing care | Arm A: hydroxychloroquine: Days 1-2: Loading phase - 400mg twice a day for 2 days Days 3 onwards: 200mg once daily, every day for 90 days (~3 months)  Arm B: hydroxychloroquine: Days 1-2: 400mg twice daily for 2 days Days 3 onwards: 400mg once a week on the same day each week (every 7th day) for 90 days (~3 months) | Placebo | Ongoing | Cambridge University Hospitals NHS Foundation Trust |
| A Study of Hydroxychloroquine and Zinc in the Prevention of COVID-19 Infection in Military Healthcare Workers^23^/COVID-Milit/Faida Ajili | NCT04377646 | - COVID-19 negative diagnosis confirmed by "rapid test" and "PCR test" (Polymerase Chain Reaction test), no clinical symptoms suggestive of COVID-19 | Hydroxychloroquine 400 mg at day 1 and day 2, then a weekly dose of 400 mg up to 2 months.  Zinc 15 mg at daily dose up to 2 months | Placebo | Not yet recruiting | Military Hospital of Tunis |
| Hydroxychloroquine and Azithromycin as Prophylaxis for Healthcare Workers Dealing With COVID19 Patients^24^/MOPHYDA/Iyad Sultan | NCT04354597 | - Age 18 to 70, Medical doctors, Nurses and Respiratory therapists caring for COVID-19 patients in ER and ICU and dedicated COVID19 units. | Hydroxychloroquine 400mg X 1 Day PO and azithromycin 500mg PO X 3 Days; weekly for 16 weeks. | No treatment | Not yet recruiting | King Hussein Cancer Center |
| Low-dose Hydroxychloroquine and Bromhexine: a Novel Regimen for COVID-19 Prophylaxis in Healthcare Professionals^25^/ELEVATE/  Julio Granados Montiel | NCT04340349 | Health Care workers with high exposure to COVID-19 patients | 200 mg of Hydroxychloroquine daily for 2 months 8 mg of Bromhexine every 8 hrs for 2 months | 8 mg of Bromhexine every 8 hrs for 2 months | Enrolling by invitation | Instituto Nacional de Rehabilitacion |
| Effect of hydroxychloroquine on prevention of covid-19 virus infection among treatment staff in Arash hospital-A double-blind clinical trial^26^/Reihaneh Pirjani | IRCT20120826010664N6 | Staffs who are in contact with patients, including nurse,physician, midwife and caregiver who have at least 3 shift a week | Hydroxychloroquine 400 mg daily and once a week for one to three months. (Based on the duration of corona virus epidemic) | Placebo |  | Tehran University of Medical Sciences |
| Effectiveness and safety of hydroxychloroquine sulfate in the preventive treatment of novel coronavirus pneumonia (COVID-19)^27^/Lu Shuihua | ChiCTR2000031174 | Healthy subjects age 18 -70 with negative COVID-19 nucleic acid test and antibody test | Hydroxychloroquine | Placebo | Pending recruitment | Shanghai Public Health Clinical Center |
| The Danish Pre-HCQ Dialysis Study:  Hydroxychloroquine for prevention of COVID-19 in dialysis-treated patients with end-stage renal disease -  A multicenter parallel-group open randomized clinical trial^28^ | 2020-001257-51 | Patients ≥18 years on chronic dialysis due to end-stage renal disease. | Hydroxychloroquine up to 200mg | Not specified | Temporarirly Halted | Rigshospitalet |
| Evaluation the effects of Hydroxychloroquine administration for COVID-19 prophylaxis^29^/HOPEC/Omidvar Rezaiemirghaed | IRCT20130917014693N10 | Age over 18 years, close contact with COVID-19 patient at least in past 4 days | Hydroxychloroquine 200 mg TDS up to 7 days | No intervention |  | Shahid Beheshti University of Medical Sciences |
| The prophylactic effect of oral hydroxy-chloroquine in close contacts of COVID-19 patients^30^/Ramin Rezaee | IRCT20200513047426N1 | Close contact with a person with COVID-19 who has been diagnosed positive with a definitive test. Close contact: contact, hand shake and talking at least once in the last 2 days for less than 2 meters with COVID-19 patient who have been diagnosed positive with a definitive test. Family, friends, relatives who have been in the same place for a period of 4 hours or more in the last 2 days with a COVID-19 patient who have been diagnosed with a definitive test . Medical staff, including physicians, nurses, midwives, and service personnel which have been in contact with COVID-19 patient who has been diagnosed with a definitive test in the past three days. | Hydroxychloroquine 400 mg tablet once on the first night at 22:002- hydroxychloroquine 200 mg tablet once a night for 5 nights at 22:00 | Placebo | Completed | Boushehr University of Medical Sciences |
| A prospective, randomized, open-label, controlled clinical study to evaluate the preventive effect of hydroxychloroquine on close contacts after exposure to the Novel Coronavirus Pneumonia (COVID-19)^31^/Zhan Zhang | ChiCTR2000029803 | Age 18 to 60 who are in close contact with suspected cases, confirmed cases, and positive test of new coronavirus nucleic acid, and those who have not taken effective protection, that is, close contacts | Hydroxychloroquine small dose, hydroxychloroquine high dose | Abidol hydrochloride small dose & high dose | Not yet recruiting | Renmin Hospital of Wuhan University |
| Preventing COVID-19 in Healthcare Workers With HCQ: A RCT ^32^/Adam Singer | NCT04347889 | - Healthcare worker at risk of Covid-19 | Oral loading dose of 800 mg followed by once weekly oral hydroxychloroquine 400 mg for 3 months | Oral Vitamin C 1,000 mg daily for three months | Not yet recruiting | Stony Brook University |
| Multi-Site, Randomized, Open-Label, Parallel-Group, Placebo-Controlled Study to Assess the Chemoprophylactic Efficacy of Chloroquine Against SARS-CoV-2/COVID-19 in Healthcare Workers at High-Risk of Exposure^33^/Scott Hahn | ACTRN12620000417987 | Age 18 to 64 years inclusive, in general good health equivalent to Army Medical Employment Classification J22 or above. Civilian personnel would need to be in good general health as advised by their General Practitioner (GP), not previously diagnosed with COVID-19; Participant works in/in association with a healthcare facility or other high-risk environment characterised by high level of contact with persons thought likely to be infected with respiratory viruses | \| Oral 500mg chloroquine phosphate tablets prophylactic weekly 10 week trial period followed with plasma chloroquine levels. \| \| --- \| | Not specified | Recruiting | Australian Defence Force Malaria and Infectious Disease Institute |
| COVID-19 PEP- High-risk Individuals in Long-term and Specialized Care - Canada^34^/Michael J Borrie | NCT04397328 | Age over 40 with two or more high-risk comorbidities that have been found to confer a higher risk of mortality. Patient/resident in an Institute (to include a rehabilitation, long term care facility, mental health facility or veteran's care) that provides bed-based care in shared semi-private or ward rooms (i.e. two or more to a room) with a patient with confirmed COVID-19 for at least 6 hours in the absence of contact and droplet precautions. Exposure with a documented or suspected COVID-19 case or from a symptomatic ( defined as common symptoms of COVID-19 including but not limited to fever, lethargy, dry cough, shortness of breath) health care worker providing direct patient contact within 3 feet without a mask for > 15min or any physical contact with the staff. Exposure may occur in single or shared bedrooms. Exposure may occur in a common dining or activity or sitting area. Any patient sharing a room or within 3 feet for > 15min or any physical contact without a mask will be considered as a contact. Patients or staff are considered as infectious for 48hrs before any symptoms onset and until masked or cleared by 2 negative swabs | Hydroxychloroquine 400mg orally once, followed in 8 hours by 400mg, then 200mg twice a day for 4 consecutive days (5 days in total)  Modified Dose 400mg orally once, followed in 8 hours by 400mg, then 200mg once a day for 4 consecutive days (5 days in total) | Placebo | Not yet recruiting | Lawson Health Research Institute |
| Clinical trial randomized, unblinded and controled for evaluation of efficacy and safety of hydroxychloroquine chemoprophylaxis against SARS-CoV-2 (COVID-19) infection in healthcare professionals.^35^/Emilio Garcia Cabrera | 2020-001421-31 | Age 18 or older, absence of SARS-CoV-2 infection (COVID-19) due to the absence of a symptoms of acute respiratory infection or a diagnostic test with a negative result, health care workers active at the center. | Hydroxychloroquine sulphate | Not specified | Ongoing | Sociedad Española de Farmacia Hospitalaria |
| Hydroxychloroquine Post Exposure Prophylaxis for Coronavirus Disease (COVID-19)^36^/Jon T. Giles | NCT04318444 | - Household contact of index case: currently residing in the same household as an individual evaluated at NYP via outpatient, emergency department (ED), or inpatient services who (1) test positive for COVID-19, or (2) are defined as suspected cases, or persons under investigations (PUI), by the treating physician. | Hydroxychloroquine 400mg twice daily on day 1; days 2-5 200mg twice daily. | Placebo | Recruiting | Columbia University |
| A Study of Hydroxychloroquine, Vitamin C, Vitamin D, and Zinc for the Prevention of COVID-19 Infection^38^/HELPCOVID-19/Sabine Hazan | NCT04335084 | Age 18 years of age or older considered to be high-risk individuals.  High-risk individuals are defined as all health care workers in hospitals, clinics, and emergency rooms, and medical facilities. | Hydroxychloroquine  Vitamin C  Vitamin D  Zinc | Placebo | Recruiting | ProgenaBiome |
| Comparative effectiveness of Chloroquine and Vitamin C prophylaxis in household contacts of confirmed COVID-19 patients^39^/ProphylaxisCOVID19/Suvimol Niyomnaitham | TCTR20200404004 | Age 18 or older, at least one household member diagnosed with COVID-19 by RT-PCR or serology within the past 7 calendar days | Chloroquine 10 mg base/kg once a day on Day 1 and Day 8 | Vitamin C 1000 mg once a day from Day 1 to Day 14 | Not yet recruiting | Health Systems Research Institute (HSRI) |
| HCQ for Primary Prophylaxis Against COVID19 in Health-care Workers^40^/Matthias Vossen | NCT04336748 | Health-care worker age 18 or older with frequent contact with confirmed COVID-19 patients, no active or past infection with SARS-CoV-2 | Hydroxychloroquine 200mg once daily for 4 weeks | Placebo | Not yet recruiting | Medical University of Vienna |
| Chemoprophylaxis of SARS-CoV-2 Infection (COVID-19) in Exposed Healthcare Workers^41^/COVIDAXIS/Elisabeth Botelho-Nevers | NCT04328285 | Adult healthcare workers involved at the time of enrolment in the care and the management of patients with confirmed or suspected SARS-CoV-2 infection in hospital settings, in outpatient care settings or in geriatric long-term care facilities. These HCWs have prolonged or repeated close contact to these patients. HCW tested negative for HIV | Hydroxychloroquine 400mg po on the evening at Day 1 and 400mg on the morning at Day 2 and 200mg once daily afterwards  Lopinavir and ritonavir200/50 mg, 2 tablets twice daily | Placebo | Active, not recruiting | Centre Hospitalier Universitaire de Saint Etienne |
| Safety And Efficacy Of Hydroxychloroquine For At Risk Population (SHARP) Against COVID-19^42^/SHARP COVID-19/Rupesh Agrawal | NCT04342156 | Aged 18 to 80 years, history of close contact or exposure to positive COVID-19 cases in the same household.  Absence of symptoms resembling COVID-19 (e.g., fever and acute respiratory or gastrointestinal symptoms) for two weeks prior to enrolment for the study. | Hydroxychloroquine sulfate. Dose: 800 milligrams in two divided doses on day 1 followed by 400mg in two divided doses on day 2, 3,4, 5. | No intervention. | Not yet recruiting | Tan Tock Seng Hospital |
| Hydroxychloroquine as Chemoprevention for COVID-19 for High Risk Healthcare Workers^43^/Jawad Kirmani | NCT04345653 | Volunteers ages 18 to 99 years, considered high-risk healthcare care providers in a hospital setting with active exposure to COVID-19 infection.  High-risk HCP's are defined as those actively working during the study duration in the Emergency Department and in the Intensive Care Setting, for the purpose of this study. | Hydroxychloroquine 400mg by mouth 6-12 hours apart on day 1, followed by 3 weeks of weekly 400mg by mouth | Not specified | Active, not recruiting | Hackensack Meridian Health |
| Hydroxychloroquine as Post-Exposure Prophylaxis Against COVID-19 Infection^44^/Susan Hoover | NCT04372017 | Age ≥ 18 years old. Employee of healthcare organization in South Dakota or Sanford Health employee in any location and with exposure to a person with COVID-19 within the last 5 days  Occupational exposure as determined by the participant's employee health department (i.e. not wearing the proper Personal Protective Equipment (PPE))  Criteria according to Center for Disease Control (CDC) guidelines  Community exposure (within 6 feet for at least 15 minutes)  No current symptoms attributable to COVID-19, per HCW report (fever, cough, difficulty breathing, sore throat)  No prior COVID-19 positive diagnosis (eligible if previous testing is negative and meets all other inclusion and exclusion) | Hydroxychloroquine 800mg on day 1 followed by 400mg on days 2-5. | Placebo | Active, not recruiting | Sanford Health |
| Prophylaxis of COVID-19 infection with hydroxychloroquine in healthcare  personnel with high risk of infection.^45^ | 2020-001536-98 | Age over 18, not having previously presented or at the time of inclusion SARS-CoV-2 infection. Healthcare professionals with high or low risk of exposure to patients with COVID-19. | Hydroxychloroquine 5-200mg | Not specified | Prematurely ended | Hospital Universitari Mutua Terrassa |
| Hydroxychloroquine as Prophylaxis for COVID-19 in Healthcare Workers^46^/HCQPreP/Ann D Chauffe | NCT04363450 | Age ≥ 18 years, Healthcare or Hospital Worker who has direct patient contact | Hydroxychloroquine loading dose will be given as 400mg for two doses 12 hours apart. This will then be followed by maintenance dosing of 200mg twice weekly for the remainder of the trial. | Placebo | Recruiting | Louisiana State University Health Sciences Center in New Orleans |
| The prophylactic effect of Hydroxychloroquine on Novel Corona virus (COVID-19) in health care providers^47^/Ramin Parvizrad | IRCT20151222025660N2 | People working in medical centers in Arak city | Hydroxychloroquine 400 mg of hydroxychloroquine a week for eight weeks | Routine care | Recruitment complete | Arak University of Medical Sciences |
| Pilot, double-blind clinical trial to evaluate the efficacy and safety of pre-exposure use of hydroxychloroquine versus placebo in the prevention of SARS-CoV-2 (COVID-19) infection in healthcare personnel.^48^/PrevenCOVID-19 | 2020-001440-26 | Adults age 18-64 | Hydroxychloroquine | Placebo | Ongoing | Fundación Pública Andaluza para la Gestión de la Investigación en Salud de Sevilla (FISEVI) |
| Treatment of non-severe confirmed cases of COVID-19 and chemoprophylaxis of their contacts as prevention strategy: a Cluster Randomized Clinical Trial^49^/PEP CoV-2 Study | 2020-001031-27 | Patients aged ≥18 who meet the definition of a contact according to the Catalan Public Health Department Guidelines | Darunavir  Cobicistat  Hydroxychloroquine | Not specified | Ongoing | Fundacion FLS De Lucha Contra El Sida Las Enfermedades Infecciosas Y La Promocion De La Salud Y La Ciencia |
