# Supplementary material for: The efficacy and safety of hydroxychloroquine for COVID-19 prophylaxis: A systematic review and meta-analysis of randomized trials
Source: PLoS One. 2021 Jan 6;16(1):e0244778. doi: 10.1371/journal.pone.0244778 (PMC7787432; doi:10.1371/journal.pone.0244778)
Supplement: S6 Table — (DOCX) [file pone.0244778.s011.docx]

S6 Table: Ineligible articles (excluding those listed in Appendix table 5)

| Number | Reference | Reason for exclusion |
| --- | --- | --- |
| 1 | Safety of Hydroxychloroquine among Outpatient Clinical Trial Participants for COVID-19  Lofgren SMM, Nicol MR, Bangdiwala AS, Pastick KA, Okafor EC, Skipper CP, Pullen MF, Engen NW, Abassi M, Williams DA, Nascene AA, Axelrod ML, Lother SA, MacKenzie LJ, Drobot G, Marten N, Cheng MP, Zarychanshi R, Schwartz IS, Silverman M, Chagla Z, Kelley LE, McDonald EG, Lee TC, Hullsiek KH, Boulware DR, Rajasingham R  First Published 2020 | Duplicate |
| 2 | Post-exposure prophylaxis or pre-emptive therapy for severe acute respiratory syndrome coronavirus 2 (SARS-CoV-2): study protocol for a pragmatic randomized-controlled trial  Sylvain A. Lother, Mahsa Abassi, Alyssa Agostinis, Ananta S. Bangdiwala, Matthew P. Cheng, Glen Drobot, Nicole Engen, Kathy H. Hullsiek, Lauren E. Kelly, Todd C. Lee, Sarah M. Lofgren, Lauren J. MacKenzie, Nicole Marten, Emily G. McDonald, Elizabeth C. Okafor, Katelyn A. Pastick, Matthew F. Pullen, Radha Rajasingham, Ilan Schwartz, Caleb P. Skipper | Duplicate |
| 3 | Boulware, D. R.; Pullen, M. F.; Bangdiwala, A. S.; Pastick, K. A.; Lofgren, S. M.; Okafor, E. C.; Skipper, C. P.; Nascene, A. A.; Nicol, M. R.; Abassi, M.; Engen, N. W.; Cheng, M. P.; LaBar, D.; Lother, S. A.; MacKenzie, L. J.; Drobot, G.; Marten, N.; Zarychanski, R.; Kelly, L. E.; Schwartz, I. S.; McDonald, E. G.; Rajasingham, R.; Lee, T. C.; Hullsiek, K. H.  A Randomized Trial of Hydroxychloroquine as Postexposure Prophylaxis for Covid-19  The New England journal of medicine. 2020;03:2020 | Duplicate |
| 4 | Mitja O, Ubals M. Corbacho M. Alemany A. Suner C. Tebe C. Tobias A. Penafiel J. Ballana E. Perez C. A. Admella P. Riera-Marti N. Laporte P. Mitja J. Clua M. Bertran L. Gavilan S. Ara J. Sarquella M. Argimon J. M. Cuatrecasas G. Canadas P. Elizalde-Torrent A. Fabregat R. Farre M. Forcada A. Flores-Mateo G. Lopez C. Muntada E. Nadal N. Narejos S. Gil-Ortega A. N. Prat N. Puig J. Quinones C. Ramirez-Viaplana F. Reyes-Uruena J. Riveira-Munoz E. Ruiz L. Sanz S. Sentis A. Sierra A. Velasco C. Vivanco-Hidalgo R. M. Zamora J. Casabona J. Vall-Mayans M. G. Beiras C. Clotet B.  A cluster-randomized trial of hydroxychloroquine AS prevention of COVID-19 transmission and disease  medRxiv 2020:2020.07.20.20157651 | Duplicate |
| 5 | https://www.isglobal.org/en/-/pre-exposure-prophylaxis-with-hydroxychloroquine-for-high-risk-healthcare-workers-during-the-covid-19-pandemic | Duplicate |
| 6 | https://clinicaltrials.gov/ct2/show/NCT04331834 | Duplicate |
| 7 | Effect of hydroxychloroquine on prevention of COVID-19 virus infection among healthcare professionals: a structured summary of a study protocol for a randomised controlled trial.  Pirjani R1, Soori T2, Dehpour AR3, Sepidarkish M4, Moini A1, Shizarpour A5, Mohammad Jafari R6 | Duplicate |
| 8 | Iran University of Medical, Sciences  Prevention of COVID-19 disease after contact with an infected patient with coronavirus after taking hydroxychloroquine in the community | Incorrect study population |
| 9 | https://clinicaltrials.gov/ct2/show/NCT04350450 | Incorrect study population |
| 10 | NCT04352933 | Duplicate |
| 11 | https://clinicaltrials.gov/ct2/show/record/NCT04308668 | Duplicate |
| 12 | Hospital Universitari MasTUA TERRASSA  Prophylaxis of COVID-19 infection with hydroxychloroquine in healthcare | Could not identify study |
| 13 | https://en.irct.ir/trial/48236 | Duplicate |
| 14 | #299 - ShanghaiPublicHealthClinical 2020  Shanghai Public Health Clinical, Center  Study for using sulfate in the prevention and control of novel coronavirus pneumonia (COVID-19) in high and low prevalence communities | Incorrect design |
| 15 | Shanghai Public Health Clinical, Center  Effectiveness and safety of hydroxychloroquine sulfate in the preventive treatment of novel coronavirus pneumonia (COVID-19) | Incorrect study population |
| 16 | https://apps.who.int/trialsearch/Trial2.aspx?TrialID=ISRCTN99916292 | Wrong intervention |
| 17 | https://clinicaltrials.gov/ct2/show/NCT04363827 | Duplicate |
| 18 | https://covid-19.cochrane.org/studies/crs-14076108 | Duplicate |
| 19 | https://clinicaltrials.gov/ct2/show/NCT04304053 | Duplicate |
| 20 | https://clinicaltrials.gov/ct2/show/NCT04331834 | Duplicate |
| 21 | https://clinicaltrials.gov/ct2/show/record/NCT04374942 | Duplicate |
| 22 | https://clinicaltrials.gov/ct2/show/NCT04352933 | Duplicate |
| 23 | NCT04304053 | Duplicate |
| 24 | NCT04329923 | Duplicate |
| 25 | NCT04346667 | Duplicate |
