# Supplementary material for: The efficacy and safety of hydroxychloroquine for COVID-19 prophylaxis: A systematic review and meta-analysis of randomized trials
Source: PLoS One. 2021 Jan 6;16(1):e0244778. doi: 10.1371/journal.pone.0244778 (PMC7787432; doi:10.1371/journal.pone.0244778)
Supplement: S7 Table — (DOCX) [file pone.0244778.s012.docx]

S7 Table: Risk of bias justifications

| COVID-19 positive | | | | | | |
| --- | --- | --- | --- | --- | --- | --- |
| **Study** | **Abella** | **Boulware** | **Mitja** | | **Rajasingham** |  |
| Random sequence generation | Low-Computer generated random number table | Low-Statistician generated randomization | Low-Cluster randomization, Table 1 demonstrates fairly uniform baseline characteristics | | Low-Computer generated algorithm. Table 1 demonstrated fairly uniform baseline characteristics |  |
| Allocation concealment | Low-Central allocation using sealedenvelope.com | Low-Central allocation | Low-Central allocation | | Low-Central allocation in the pharmacy sing a permuted block sequence |  |
| Blinding of participants and personnel | Low-Placebo controlled, double blind, and objective outcome | Low-Placebo controlled, double blind, and objective outcome | Low-Open label, non-placebo controlled, however objective outcome as needed one symptom consistent with COVID-19 as well as a positive SARS-CoV-2 RT-PCR test | | Low-Placebo controlled, double blind. Although the outcome was either symptoms consistent with COVID-19 or a positive SARS-COV-2 RT-PCR test |  |
| Blinding of outcome assessment | Low-Placebo controlled, double blind, and objective outcome | Low-Placebo controlled, double blind, and objective outcome | Low-Open label, non-placebo controlled, however objective outcome as needed one symptom consistent with COVID-19 as well as a positive SARS-CoV-2 RT-PCR test | | Low-Placebo controlled, double blind. Although the outcome was either symptoms consistent with COVID-19 or a positive SARS-COV-2 RT-PCR test |  |
| Incomplete outcome data | Low-Only 5% of randomized patients were not evaluated, 5 in the intervention group and 2 in the control group (therefore fairly balanced). Intention-to-treat analysis performed | Low- 9% loss to follow up, 4 withdrew in each arm. Intention-to-treat analysis performed | Low-6.4% were excluded from the ITT analysis secondary to missing results or screening logs | | Low-Only 0.5% were excluded from the ITT analysis after being excluded from the study post randomization for failing to make inclusion/exclusion criteria. |  |
| Selective reporting | Low-Outcome was pre-defined on clinicaltrials.gov and in the protocol | Low-All outcomes reported | Low-Pre-defined outcome on clincialtrials.gov | | Low-All outcomes reported |  |
| Other bias | Low-Stopped early for futility due to reduced recruitment rate secondary to tapering number of COVID-19 cases. In addition, the funding agent had no role in the design, conduct, or analysis of the study, and no role in the manuscript preparation | Low- Stopped early for futility. Funding from the government | Low- Funding from crowd sourcing and industry. Industry had no role in the conduct of the trial, analysis, or decision to submit the manuscript | | Low- Stopped early for futility due to reduced recruitment rate secondary to tapering number of COVID-19 cases. Funding from the government therefore unlikely to bias trial results |  |
| Overall risk | Low risk | Low risk | Low risk | | Low risk |  |
| PCR positive | | | | | |  |
| **Study** | **Abella** | **Boulware** | **Mitja** | | **Rajasingham** |  |
| Random sequence generation | Low-Computer generated random number table | Low-Statistician generated randomization | Low-Cluster randomization, Table 1 demonstrates fairly uniform baseline characteristics | | Low-Computer generated algorithm. Table 1 demonstrated fairly uniform baseline characteristics |  |
| Allocation concealment | Low-Central allocation using sealedenvelope.com | Low-Central allocation | Low-Central allocation | | Low-Central allocation in the pharmacy sing a permuted block sequence |  |
| Blinding of participants and personnel | Low-Placebo controlled, double blind, and objective outcome | Low-Placebo controlled, double blind, and objective outcome | Low-Open label, non-placebo controlled, however objective outcome of a positive SARS-CoV-2 RT-PCR test | | Low-Placebo controlled, double blind, and objective outcome |  |
| Blinding of outcome assessment | Low-Placebo controlled, double blind, and objective outcome | Low-Placebo controlled, double blind, and objective outcome | Low-Open label, non-placebo controlled, however objective outcome | | Low-Placebo controlled, double blind, and objective outcome |  |
| Incomplete outcome data | Low-Only 5% of randomized patients were not evaluated, 5 in the intervention group and 2 in the control group (therefore fairly balanced). Intention-to-treat analysis performed | Low- 9% loss to follow up, 4 withdrew in each arm. Intention-to-treat analysis performed | Low-6.4% were excluded from the ITT analysis secondary to missing results or screening logs | | Low-Only 0.5% were excluded from the ITT analysis after being excluded from the study post randomization for failing to make inclusion/exclusion criteria. |  |
| Selective reporting | Low-Outcome was pre-defined on clinicaltrials.gov and in the protocol | Low-All outcomes reported | Low-Pre-defined outcome on clincialtrials.gov | | Low-All outcomes reported |  |
| Other bias | Low-Stopped early for futility due to reduced recruitment rate secondary to tapering number of COVID-19 cases. In addition, the funding agent had no role in the design, conduct, or analysis of the study, and no role in the manuscript preparation | Low- Stopped early for futility. Funding from the government | Low- Funding from crowd sourcing and industry. Industry had no role in the conduct of the trial, analysis, or decision to submit the manuscript | | Low- Stopped early for futility due to reduced recruitment rate secondary to tapering number of COVID-19 cases. Funding from the government therefore unlikely to bias trial results |  |
| Overall risk | Low risk | Low risk | Low risk | | Low risk |  |
| Hospitalization | | | | | | |
| **Study** | **Abella** | **Boulware** | **Mitja** | | **Rajasingham** |  |
| Random sequence generation | Low-Computer generated random number table | Low-Statistician generated randomization | Low-Cluster randomization, Table 1 demonstrates fairly uniform baseline characteristics | | Low-Computer generated algorithm. Table 1 demonstrated fairly uniform baseline characteristics |  |
| Allocation concealment | Low-Central allocation using sealedenvelope.com | Low-Central allocation | Low-Central allocation | | Low-Central allocation in the pharmacy sing a permuted block sequence |  |
| Blinding of participants and personnel | Low-Placebo controlled, double blind, and objective outcome | Low-Placebo controlled, double blind, and objective outcome | Low-Open label, non-placebo controlled, however objective outcome | | Low-Placebo controlled, double blind and, objective outcome |  |
| Blinding of outcome assessment | Low-Placebo controlled, double blind, and objective outcome | Low-Placebo controlled, double blind, and objective outcome | Low-Open label, non-placebo controlled, however objective outcome | | Low-Placebo controlled, double blind and, objective outcome |  |
| Incomplete outcome data | Low-Only 5% of randomized patients were not evaluated, 5 in the intervention group and 2 in the control group (therefore fairly balanced). Intention-to-treat analysis performed | Low - 9% loss to follow up, 4 withdrew in each arm. Intention-to-treat analysis performed | Low-6.4% were excluded from the ITT analysis secondary to missing results or screening logs | | Low-Only 0.5% were excluded from the ITT analysis after being excluded from the study post randomization for failing to make inclusion/exclusion criteria. |  |
| Selective reporting | Low -Hospitalization was a pre-defined outcome on clinicaltrials.gov | Low-Outcome reported a priori | Low-Pre-defined outcome on clincialtrials.gov | | Low-All outcomes reported |  |
| Other bias | Low - Stopped early for futility due to reduced recruitment secondary to tapering number of COVID-19 cases. In addition, the funding individuals had no role in the design, conduct, or analysis of the study, and no role in the manuscript preparation | Low-Stopped early for futility. Funding from the government | Low- Funding from crowd sourcing and industry. Industry had no role in the conduct of the trial, analysis, or decision to submit the manuscript | | Low- Stopped early for futility due to reduced recruitment rate secondary to tapering number of COVID-19 cases. Funding from the government therefore unlikely to bias trial results |  |
| Overall risk | Low risk | Low risk | Low risk | | Low risk |  |
| Mortality | | | | | | |
| **Study** | **Abella** | **Boulware** | **Mitja** | | **Rajasingham** |  |
| Random sequence generation | Low- Computer generated random number table | Low -Statistician generated randomization | Low-Cluster randomization, Table 1 demonstrates fairly uniform baseline characteristics | | Low-Computer generated algorithm. Table 1 demonstrated fairly uniform baseline characteristics |  |
| Allocation concealment | Low-Central allocation using sealedenvelope.com | Low-Central allocation | Low-Central allocation | | Low-Central allocation in the pharmacy sing a permuted block sequence |  |
| Blinding of participants and personnel | Low-Placebo controlled, double blind, and objective outcome | Low -Placebo controlled, double blind, and objective outcome | Low-Open label, non-placebo controlled, however objective outcome | | Low-Placebo controlled, double blind and, objective outcome |  |
| Blinding of outcome assessment | Low-Placebo controlled, double blind, and objective outcome | Low-Placebo controlled, double blind, and objective outcome | Low-Open label, non-placebo controlled, however objective outcome | | Low-Placebo controlled, double blind and, objective outcome |  |
| Incomplete outcome data | Low-Only 5% of randomized patients were not evaluated, 5 in the intervention group and 2 in the control group (therefore fairly balanced). Intention-to-treat analysis performed | Low-9% loss to follow up, 4 withdrew in each arm. Intention-to-treat analysis performed | Low-6.4% were excluded from the ITT analysis secondary to missing results or screening logs | | Low-Only 0.5% were excluded from the ITT analysis after being excluded from the study post randomization for failing to make inclusion/exclusion criteria. |  |
| Selective reporting | Low-Mortality was a pre-defined outcome in their trial protocol | Low-Outcome reported a priori | Low-Pre-defined outcome on clincialtrials.gov | | Low-All outcomes reported |  |
| Other bias | Low-Stopped early for futility due to reduced recruitment secondary to tapering number of COVID-19 cases. In addition, the funding individuals had no role in the design, conduct, or analysis of the study, and no role in the manuscript preparation | Low-Stopped early for futility. Funding from the government | Low- Funding from crowd sourcing and industry. Industry had no role in the conduct of the trial, analysis, or decision to submit the manuscript | | Low- Stopped early for futility due to reduced recruitment rate secondary to tapering number of COVID-19 cases. Funding from the government therefore unlikely to bias trial results |  |
| Overall risk | Low risk | Low risk | Low risk | | Low risk |  |
| Adverse events | | | | | | |
| **Study** | **Abella** | **Boulware** | **Mitja** | | **Rajasingham** |  |
| Random sequence generation | Low-Computer generated random number table | Low-Statistician generated randomization | Low-Cluster randomization, Table 1 demonstrates fairly uniform baseline characteristics | | Low-Computer generated algorithm. Table 1 demonstrated fairly uniform baseline characteristics |  |
| Allocation concealment | Low-Central allocation using sealedenvelope.com | Low-Central allocation | Low-Central allocation | | Low-Central allocation in the pharmacy sing a permuted block sequence |  |
| Blinding of participants and personnel | Low-Placebo controlled, double blind | Low-Placebo controlled, double blind | High-Open label, non-placebo controlled, and “adverse events” includes very subjective outcomes such as malaise and tingling. | | Low-Placebo controlled, double blind |  |
| Blinding of outcome assessment | Low-Placebo controlled, double blind | Low-Placebo controlled, double blind | High-Open label, non-placebo controlled, and “adverse events” include very subjective outcomes such as malaise and tingling. | | Low-Placebo controlled, double blind |  |
| Incomplete outcome data | Low-Only 5% of randomized patients were not evaluated, 5 in the intervention group and 2 in the control group (therefore fairly balanced). Intention-to-treat analysis performed | Low - 9% loss to follow up, 4 withdrew in each arm. Intention-to-treat analysis performed | Low-6.4% were excluded from the ITT analysis secondary to missing results or screening logs | | Low-Only 0.5% were excluded from the ITT analysis after being excluded from the study post randomization for failing to make inclusion/exclusion criteria. |  |
| Selective reporting | Low-Adverse events was a pre-defined outcome on clinicaltrials.gov | Low-Outcome reported a priori | Low-Pre-defined outcome on clincialtrials.gov | | Low-All outcomes reported |  |
| Other bias | Low-Stopped early for futility due to reduced recruitment secondary to tapering number of COVID-19 cases. In addition, the funding individuals had no role in the design, conduct, or analysis of the study, and no role in the manuscript preparation | Low-Stopped early for futility. Funding from the government | Low- Funding from crowd sourcing and industry. Industry had no role in the conduct of the trial, analysis, or decision to submit the manuscript | | Low- Stopped early for futility due to reduced recruitment rate secondary to tapering number of COVID-19 cases. Funding from the government therefore unlikely to bias trial results |  |
| Overall risk | Low risk | Low risk | High risk | | Low risk |  |
| Nausea or dyspepsia | | | | | | |
| **Studies** | **Abella** | **Boulware** | **Rajasingham** | | |  |
| Random sequence generation | Low-Computer generated random number table | Low-Statistician generated randomization | Low-Computer generated algorithm. Table 1 demonstrated fairly uniform baseline characteristics | | |  |
| Allocation concealment | Low-Central allocation using sealedenvelope.com | Low-Central allocation | Low-Central allocation in the pharmacy sing a permuted block sequence | | |  |
| Blinding of participants and personnel | Low-Placebo controlled, double blind | Low risk-Placebo controlled, double blind, and objective outcome | Low-Placebo controlled, double blind | | |  |
| Blinding of outcome assessment | Low-Placebo controlled, double blind | Low-Placebo controlled, double blind, and objective outcome | Low-Placebo controlled, double blind | | |  |
| Incomplete outcome data | Low-Only 5% of randomized patients were not evaluated, 5 in the intervention group and 2 in the control group (therefore fairly balanced). Intention-to-treat analysis performed | Low-9% loss to follow up, 4 withdrew in each arm. Intention-to-treat analysis performed | Low-Only 0.5% were excluded from the ITT analysis after being excluded from the study post randomization for failing to make inclusion/exclusion criteria. | | |  |
| Selective reporting | Low-Although not explicitly cited in the protocol or clincialtrials.gov, they did indicate adverse events was a pre-defined outcome | Low-Outcome reported a priori | Low-All outcomes reported | | |  |
| Other bias | Low-Stopped early for futility due to reduced recruitment secondary to tapering number of COVID-19 cases. In addition, the funding individuals had no role in the design, conduct, or analysis of the study, and no role in the manuscript preparation | Low-Stopped early for futility. Funding from the government | Low- Stopped early for futility due to reduced recruitment rate secondary to tapering number of COVID-19 cases. Funding from the government therefore unlikely to bias trial results | | |  |
| Overall risk | Low risk | Low risk | Low risk | | |  |
| Vomiting or diarrhea | | | | | | |
| **Study** | **Abella** | **Boulware** | **Mitja** | | **Rajasingham** |  |
| Random sequence generation | Low-Computer generated random number table | Low-Statistician generated randomization | Low-Cluster randomization, Table 1 demonstrates fairly uniform baseline characteristics | | Low-Computer generated algorithm. Table 1 demonstrated fairly uniform baseline characteristics |  |
| Allocation concealment | Low-Central allocation using sealedenvelope.com | Low-Central allocation | Low-Central allocation | | Low-Central allocation in the pharmacy sing a permuted block sequence |  |
| Blinding of participants and personnel | Low-Placebo controlled, double blind | Low-Placebo controlled, double blind | High-Open label, non-placebo controlled, and diarrhea can be very subjective | | Low-Placebo controlled, double blind |  |
| Blinding of outcome assessment | Low-Placebo controlled, double blind | Low-Placebo controlled, double blind | High-Open label, non-placebo controlled, and diarrhea can be very subjective | | Low-Placebo controlled, double blind |  |
| Incomplete outcome data | Low-Only 5% of randomized patients were not evaluated, 5 in the intervention group and 2 in the control group (therefore fairly balanced). Intention-to-treat analysis performed | Low- 9% loss to follow up, 4 withdrew in each arm. Intention-to-treat analysis performed | Low-6.4% were excluded from the ITT analysis secondary to missing results or screening logs | | Low-Only 0.5% were excluded from the ITT analysis after being excluded from the study post randomization for failing to make inclusion/exclusion criteria. |  |
| Selective reporting | Low-Although not explicitly cited in the protocol or clincialtrials.gov, they did indicate adverse events was a pre-defined outcome | Low-Outcome reported a priori | Low-Pre-defined outcome on clincialtrials.gov | | Low-All outcomes reported |  |
| Other bias | Low-Stopped early for futility due to reduced recruitment secondary to tapering number of COVID-19 cases. In addition, the funding individuals had no role in the design, conduct, or analysis of the study, and no role in the manuscript preparation | Low-Stopped early for futility. Funding from the government | Low- Funding from crowd sourcing and industry. Industry had no role in the conduct of the trial, analysis, or decision to submit the manuscript | | Low- Stopped early for futility due to reduced recruitment rate secondary to tapering number of COVID-19 cases. Funding from the government therefore unlikely to bias trial results |  |
| Overall risk | Low risk | Low risk | High risk | | Low risk |  |
| Arrhythmia | | | | | | |
| **Study** | **Abella** | **Boulware** | **Mitja** | | **Rajasingham** |  |
| Random sequence generation | Low-Computer generated random number table | Low-Statistician generated randomization | Low-Cluster randomization, Table 1 demonstrates fairly uniform baseline characteristics | | Low-Computer generated algorithm. Table 1 demonstrated fairly uniform baseline characteristics |  |
| Allocation concealment | Low-Central allocation using sealedenvelope.com | Low-Central allocation | Low-Central allocation | | Low-Central allocation in the pharmacy sing a permuted block sequence |  |
| Blinding of participants and personnel | Low-Placebo controlled, double blind | Low-Placebo controlled, double blind, and objective outcome | High-Open label, non-placebo controlled, and part of their definition of this outcome included “palpitations” | | Low-Placebo controlled, double blind |  |
| Blinding of outcome assessment | Low-Placebo controlled, double blind | Low-Placebo controlled, double blind, and objective outcome | High-Open label, non-placebo controlled, and part of their definition of this outcome included “palpitations” | | Low-Placebo controlled, double blind |  |
| Incomplete outcome data | Low-Only 5% of randomized patients were not evaluated, 5 in the intervention group and 2 in the control group (therefore fairly balanced). Intention-to-treat analysis performed | Low-9% loss to follow up, 4 withdrew in each arm. Intention-to-treat analysis performed | Low-6.4% were excluded from the ITT analysis secondary to missing results or screening logs | | Low-Only 0.5% were excluded from the ITT analysis after being excluded from the study post randomization for failing to make inclusion/exclusion criteria. |  |
| Selective reporting | Low-Although not explicitly cited in the protocol or clincialtrials.gov, they did indicate adverse events was a pre-defined outcome | Low-Outcome reported a priori | Low-Pre-defined outcome on clincialtrials.gov | | Low-All outcomes reported |  |
| Other bias | Low-Stopped early for futility due to reduced recruitment secondary to tapering number of COVID-19 cases. In addition, the funding individuals had no role in the design, conduct, or analysis of the study, and no role in the manuscript preparation | Low- Stopped early for futility. Funding from the government | Low- Funding from crowd sourcing and industry. Industry had no role in the conduct of the trial, analysis, or decision to submit the manuscript | | Low- Stopped early for futility due to reduced recruitment rate secondary to tapering number of COVID-19 cases. Funding from the government therefore unlikely to bias trial results |  |
| Overall risk | Low risk | Low risk | High risk | | Low risk |  |
| Visual changes | | | | | | |
| **Study** | **Boulware** | **Rajasingham** | | | | |
| Random sequence generation | Low risk-Statistician generated randomization | Low-Computer generated algorithm. Table 1 demonstrated fairly uniform baseline characteristics | | | | |
| Allocation concealment | Low-Central allocation | Low-Central allocation in the pharmacy sing a permuted block sequence | | | | |
| Blinding of participants and personnel | Low-Placebo controlled, double blind, and objective outcome | Low-Placebo controlled, double blind, and objective outcome | | | | |
| Blinding of outcome assessment | Low-Placebo controlled, double blind, and objective outcome | Low-Placebo controlled, double blind, and objective outcome | | | | |
| Incomplete outcome data | Low- 9% loss to follow up, 4 withdrew in each arm. Intention-to-treat analysis performed | Low-Only 0.5% were excluded from the ITT analysis after being excluded from the study post randomization for failing to make inclusion/exclusion criteria. | | | | |
| Selective reporting | Low-Outcome reported a priori | Low-All outcomes reported | | | | |
| Other bias | Low-Stopped early for futility. Funding from the government | Low- Stopped early for futility due to reduced recruitment rate secondary to tapering number of COVID-19 cases. Funding from the government therefore unlikely to bias trial results | | | | |
| Overall risk | Low risk | Low risk | | | | |
| Compliance | | | | | | |
| **Study** | **Abella** | **Boulware** | | **Mitja** | | |
| Random sequence generation | Low-Computer generated random number table | Low-Statistician generated randomization | | Low-Cluster randomization, Table 1 demonstrates fairly uniform baseline characteristics | | |
| Allocation concealment | Low-Central allocation using sealedenvelope.com | Low-Central allocation | | Low-Central allocation | | |
| Blinding of participants and personnel | Low-Placebo controlled, double blind, and objective outcome | Low-Placebo controlled, double blind, and objective outcome | | High-Open label, non-placebo controlled, and compliance could be heavily influenced by knowing your allocation | | |
| Blinding of outcome assessment | Low-Placebo controlled, double blind, and objective outcome | Low-Placebo controlled, double blind, and objective outcome | | Low-Open label, non-placebo controlled, however assessment of compliance would be objective to assess | | |
| Incomplete outcome data | Low-Only 5% of randomized patients were not evaluated, 5 in the intervention group and 2 in the control group (therefore fairly balanced). Intention-to-treat analysis performed | Low- 9% loss to follow up, 4 withdrew in each arm. Intention-to-treat analysis performed | | Low-6.4% were excluded from the ITT analysis secondary to missing results or screening logs | | |
| Selective reporting | Low-Compliance as an outcome was reported in the protocol | Low-Outcome reported a priori | | Low-Pre-defined outcome on clincialtrials.gov | | |
| Other bias | Low-Stopped early for futility due to reduced recruitment secondary to tapering number of COVID-19 cases. In addition, the funding individuals had no role in the design, conduct, or analysis of the study, and no role in the manuscript preparation | Low-Stopped early for futility. Funding from the government | | Low- Funding from crowd sourcing and industry. Industry had no role in the conduct of the trial, analysis, or decision to submit the manuscript | | |
| Overall risk | Low risk | Low risk | | High risk | | |
