# Supplementary material for: The efficacy and safety of hydroxychloroquine for COVID-19 prophylaxis: A systematic review and meta-analysis of randomized trials
Source: PLoS One. 2021 Jan 6;16(1):e0244778. doi: 10.1371/journal.pone.0244778 (PMC7787432; doi:10.1371/journal.pone.0244778)
Supplement: S8 Table — (DOCX) [file pone.0244778.s013.docx]

S8 Table: Summary of findings table

| **Certainty assessment** | | | | | | | **№ of patients** | | **Effect** | | **Certainty** | **Importance** |
| --- | --- | --- | --- | --- | --- | --- | --- | --- | --- | --- | --- | --- |
| **№ of studies** | **Study design** | **Risk of bias** | **Inconsistency** | **Indirectness** | **Imprecision** | **Other considerations** | **Hydroxychloroquine** | **placebo** | **Relative (95% CI)** | **Absolute (95% CI)** |  |  |
| **COVID-19 positive** | | | | | | | | | | | | |
| 4 | randomised trials | not serious | not serious | not serious | serious ^a^ | none | 130/1785 (7.3%) | 122/1309 (9.3%) | **RR 0.82** (0.65 to 1.04) | **17 fewer per 1,000** (from 33 fewer to 4 more) | ⨁⨁⨁◯ MODERATE | CRITICAL |
| **PCR positive** | | | | | | | | | | | | |
| 4 | randomised trials | not serious | not serious | not serious | serious ^b^ | none | 45/1783 (2.5%) | 42/1311 (3.2%) | **RR 0.97** (0.64 to 1.47) | **1 fewer per 1,000** (from 12 fewer to 15 more) | ⨁⨁⨁◯ MODERATE | CRITICAL |
| **Mortality** | | | | | | | | | | | | |
| 4 | randomised trials | not serious | not serious | not serious | very serious ^c^ | none | 1/1783 (0.1%) | 0/1311 (0.0%) | **RR 3.26** (0.13 to 79.74) | **0 fewer per 1,000** (from 0 fewer to 0 fewer) | ⨁⨁◯◯ LOW | CRITICAL |
| **Hospitalizations** | | | | | | | | | | | | |
| 4 | randomised trials | not serious | not serious | not serious | serious ^d^ | none | 15/1783 (0.8%) | 13/1311 (1.0%) | **RR 0.72** (0.34 to 1.50) | **3 fewer per 1,000** (from 7 fewer to 5 more) | ⨁⨁⨁◯ MODERATE | CRITICAL |
| **Compliance** | | | | | | | | | | | | |
| 3 | randomised trials | not serious | serious ^e^ | not serious | serious ^f^ | none | 670/800 (83.8%) | 728/818 (89.0%) | **RR 0.95** (0.89 to 1.02) | **44 fewer per 1,000** (from 98 fewer to 18 more) | ⨁⨁◯◯ LOW | IMPORTANT |
| **At least 1 side effect** | | | | | | | | | | | | |
| 4 | randomised trials | not serious | serious ^g^ | not serious | not serious | none | 666/1719 (38.7%) | 197/1259 (15.6%) | **RR 2.76** (1.38 to 5.55) | **275 more per 1,000** (from 59 more to 712 more) | ⨁⨁⨁◯ MODERATE | IMPORTANT |
| **Nausea or dyspepsia** | | | | | | | | | | | | |
| 3 | randomised trials | not serious | serious ^h^ | not serious | not serious | none | 259/1397 (18.5%) | 89/909 (9.8%) | **RR 1.91** (1.10 to 3.31) | **89 more per 1,000** (from 10 more to 226 more) | ⨁⨁⨁◯ MODERATE | IMPORTANT |
| **Vomiting or diarrhea** | | | | | | | | | | | | |
| 4 | randomised trials | not serious | serious ^i^ | not serious | not serious | none | 379/1719 (22.0%) | 67/1259 (5.3%) | **RR 4.60** (1.78 to 11.91) | **192 more per 1,000** (from 42 more to 581 more) | ⨁⨁⨁◯ MODERATE | IMPORTANT |
| **Vision changes** | | | | | | | | | | | | |
| 2 | randomised trials | not serious | not serious | serious ^j^ | serious ^k^ | none | 14/1332 (1.1%) | 3/844 (0.4%) | **RR 2.27** (0.70 to 7.29) | **5 more per 1,000** (from 1 fewer to 22 more) | ⨁⨁◯◯ LOW | IMPORTANT |
| **Arrhythmia** | | | | | | | | | | | | |
| 4 | randomised trials | not serious | not serious | serious ^l^ | serious ^m^ | none | 11/1719 (0.6%) | 8/1259 (0.6%) | **RR 0.71** (0.29 to 1.73) | **2 fewer per 1,000** (from 5 fewer to 5 more) | ⨁⨁◯◯ LOW | IMPORTANT |

**CI:** Confidence interval; **RR:** Risk ratio

#### Explanations

a. Fewer than 300 events. Estimate of effect ranges from 33 fewer to 4 more events

b. Fewer than 300 events. Estimate of effect ranged from 12 fewer to 15 more events

c. Extremely small number of total events

d. Fewer than 300 events. Estimate of effect ranges from 7 fewer to 5 more events

e. Important heterogeneity suggested by an I-squared=61%, P=0.08, df=2, Chi-squared=5.10, and some confidence intervals do not overlap

f. Greater than 300 events, however estimate of effect ranges from 98 fewer to 18 more events

g. Important heterogeneity suggested by an I-squared=95%, P<0.00001, df=3, Chi-squared=59.64, different estimates of effect, and confidence intervals do not overlap

h. Important heterogeneity suggested by an I-squared=74%, P=0.02, df=2, Chi-Squared=7.63, different estimates of effect, and confidence intervals do not overlap

i. Important heterogeneity suggested by an I-squared=92%, P<0.00001, df=3, Chi-squared=36.56, different estimates of effect, and confidence intervals do not overlap

j. Visual changes vary in patient importance from mild dizziness to blindness

k. Fewer than 300 events, estimate of effect ranges from 1 fewer to 22 more events

l. The outcome of arrhythmia varies in patient importance from atrial fibrillation to ventricular tachycardia

m. Fewer than 300 events and estimate of effect ranges from 5 fewer to 5 more events
