# Supplementary material for: The efficacy and safety of hydroxychloroquine for COVID-19 prophylaxis: A systematic review and meta-analysis of randomized trials
Source: PLoS One. 2021 Jan 6;16(1):e0244778. doi: 10.1371/journal.pone.0244778 (PMC7787432; doi:10.1371/journal.pone.0244778)
Supplement: S9 Table — (DOCX) [file pone.0244778.s014.docx]

S9 Table: Summary table of subgroup analyses

| **Outcome** | **Subgroup analysis performed** | **Estimate of effect and 95% CI** | **P-interaction** |
| --- | --- | --- | --- |
| Disease transmission | Daily dosing | RR 0.87 (0.65 to 1.17) | 0.54 |
|  | Weekly dosing | RR 0.75 (0.50 to 1.10) |  |
|  | Pre-exposure prophylaxis | RR 0.75 (0.50 to 1.10) | 0.54 |
|  | Post-exposure prophylaxis | RR 0.87 (0.65 to 1.17) |  |
| Hospitalization | Daily dosing | RR 1.06 (0.27 to 4.22) | 0.51 |
|  | Weekly dosing | RR 0.61 (0.26 to 1.47) |  |
|  | Pre-exposure prophylaxis | RR 0.61 (0.26 to 1.47) | 0.51 |
|  | Post-exposure prophylaxis | RR 1.06 (0.27 to 4.22) |  |
| Mortality | Daily dosing | -- | -- |
|  | Weekly dosing | -- |  |
|  | Pre-exposure prophylaxis | -- | -- |
|  | Post-exposure prophylaxis | -- |  |
| Adverse event | Daily dosing | RR 3.37 (1.26 to 8.98) | 0.14 |
|  | Weekly dosing | RR 1.58 (1.30 to 1.93) |  |
| Nausea/dyspepsia | Daily dosing | RR 2.22 (0.96 to 5.11) | 0.40 |
|  | Weekly dosing | RR 1.52 (1.15 to 2.01) |  |
| Vomiting/diarrhea | Daily dosing | RR 6.23 (2.26 to 17.14) | 0.04 |
|  | Weekly dosing | RR 2.01 (1.41 to 2.86) |  |
| Arrythmia | Daily dosing | RR 3.26 (0.13 to 79.74) | 0.33 |
|  | Weekly dosing | RR 0.63 (0.25 to 1.58) |  |
| Visual changes | Daily dosing | RR 7.04 (0.36 to 135.79) | 0.41 |
|  | Weekly dosing | RR 1.84 (0.52 to 6.56) |  |
| Compliance | Daily dosing | -- | -- |
|  | Weekly dosing | -- |  |
|  | Pre-exposure prophylaxis | -- | -- |
|  | Post-exposure prophylaxis | -- |  |

-- denotes unable to perform analysis due to insufficient data; CI=Confidence interval; NA=not applicable; RR=relative risk
