# Supplementary material for: The efficacy and safety of hydroxychloroquine for COVID-19 prophylaxis: A systematic review and meta-analysis of randomized trials
Source: PLoS One. 2021 Jan 6;16(1):e0244778. doi: 10.1371/journal.pone.0244778 (PMC7787432; doi:10.1371/journal.pone.0244778)
Supplement: S10 Table — (DOCX) [file pone.0244778.s015.docx]

S10 Table: Summary table of sensitivity analyses

| **Outcome** | **Subgroup analysis performed** | **Estimate of effect and 95% CI** | **I^2^** |
| --- | --- | --- | --- |
| Disease transmission | Low ROB | -- | -- |
|  | Peer reviewed | -- | -- |
|  | Full sample size analysis | RR 0.85 (0.69 to 1.03) | 0% |
| Hospitalization | Low ROB | -- | -- |
|  | Peer reviewed | -- | -- |
|  | Full sample size analysis | RR 0.80 (0.45 to 1.44) | 0% |
| Mortality | Low ROB | -- | -- |
|  | Peer reviewed | -- | -- |
|  | Full sample size analysis | 0.68 (0.22 to 2.07) | NA |
| Adverse events | Low ROB | RR 1.87 (1.39 to 2.51) | 66% |
|  | Peer reviewed | -- | -- |
|  | Full sample size analysis | RR 2.81 (1.07 to 7.37) | 98% |
| Nausea/dyspepsia | Low ROB | -- | -- |
|  | Peer reviewed | -- | -- |
|  | Full sample size analysis | -- | -- |
| Vomiting/diarrhea | Low ROB | RR 3.03 (1.57 to 5.84) | 79% |
|  | Peer reviewed | -- | -- |
|  | Full sample size analysis | RR 4.73 (1.46 to 15.32) | 96% |
| Arrythmia | Low ROB | RR 0.63 (0.25 to 1.58) | 0% |
|  | Peer reviewed | -- | -- |
|  | Full sample size analysis | RR 1.48 (0.18 to 12.25) | 71% |
| Visual changes | Low ROB | -- | -- |
|  | Peer reviewed | -- | -- |
|  | Full sample size analysis | -- | -- |
| Compliance | Low ROB | 0.92 (0.86 to 0.98) | 0% |
|  | Peer reviewed | -- | -- |
|  | Full sample size analysis | 0.95 (0.90 to 1.01) | 60% |

-- denotes unable to perform analysis due to insufficient data; CI=Confidence interval; NA=not applicable; RR=relative risk
